# Biomechanics of Human Fetal Hearts with Critical Aortic Stenosis

**DOI:** 10.1007/s10439-020-02683-x

**Published:** 2020-11-11

**Authors:** Chi Wei Ong, Meifeng Ren, Hadi Wiputra, Joy Mojumder, Wei Xuan Chan, Andreas Tulzer, Gerald Tulzer, Martin Lindsay Buist, Citra Nurfarah Zaini Mattar, Lik Chuan Lee, Choon Hwai Yap

**Affiliations:** 1 Department of Biomedical Engineering, National University of Singapore, Singapore, Singapore; 2 Department of Mechanical Engineering, Michigan State University, East Lansing, United States; 3 Department of Pediatric Cardiology, Children’s Heart Center Linz, Kepler University Hospital, Linz, Austria; 4 Department of Obstetrics and Gynecology, Yong Loo Lin School of Medicine, National University of Singapore, National University Health System, Singapore, Singapore; 5 Department of Bioengineering, Imperial College London, London, UK

**Keywords:** Fetal aortic stenosis, Evolving hypoplastic left heart syndrome, Fetal mitral regurgitation, Fetal left ventricle, Fetal heart biomechanics, Finite element method

## Abstract

Critical aortic stenosis (AS) of the fetal heart causes a drastic change in the cardiac biomechanical environment. Consequently, a substantial proportion of such cases will lead to a single-ventricular birth outcome. However, the biomechanics of the disease is not well understood. To address this, we performed Finite Element (FE) modelling of the healthy fetal left ventricle (LV) based on patient-specific 4D ultrasound imaging, and simulated various disease features observed in clinical fetal AS to understand their biomechanical impact. These features included aortic stenosis, mitral regurgitation (MR) and LV hypertrophy, reduced contractility, and increased myocardial stiffness. AS was found to elevate LV pressures and myocardial stresses, and depending on severity, can drastically decrease stroke volume and myocardial strains. These effects are moderated by MR. AS alone did not lead to MR velocities above 3 m/s unless LV hypertrophy was included, suggesting that hypertrophy may be involved in clinical cases with high MR velocities. LV hypertrophy substantially elevated LV pressure, valve flow velocities and stroke volume, while reducing LV contractility resulted in diminished LV pressure, stroke volume and wall strains. Typical extent of hypertrophy during fetal AS in the clinic, however, led to excessive LV pressure and valve velocity in the FE model, suggesting that reduced contractility is typically associated with hypertrophy. Increased LV passive stiffness, which might represent fibroelastosis, was found to have minimal impact on LV pressures, stroke volume, and wall strain. This suggested that fibroelastosis could be a by-product of the disease progression and does not significantly impede cardiac function. Our study demonstrates that FE modelling is a valuable tool for elucidating the biomechanics of congenital heart disease and can calculate parameters which are difficult to measure, such as intraventricular pressure and myocardial stresses.

**Electronic supplementary material:**

The online version of this article (10.1007/s10439-020-02683-x) contains supplementary material, which is available to authorized users.

## Introduction

Congenital aortic stenosis (AS) carries a prevalence of 0.2–0.5 per 1000 live births.^14^ It can develop during mid-gestation leading to severe cardiac dysfunction, and a substantial proportion of affected fetuses will develop hypoplastic left heart syndrome by birth.^30^ Aortic stenosis causes left ventricular outflow track obstruction and elevated left ventricular (LV) pressures,^9^ which in turn elevates myocardial stresses. Mitral regurgitation (MR) is also frequently present.^1^,^11^ LV outflow obstruction decreases stroke volume and the extent of myocardial strain, and can do so severely.^24^ The LV may develop hypertrophy^31^ and endocardial fibroelastosis.^32^ In critical AS, fetal aortic valvuloplasty can be used as an early intrauterine intervention to relieve the outflow obstruction, with recent studies reporting high technical success rates of up to 94% 16 with a 59–73% likelihood of biventricular function at birth.^16^,^17^ This is a significant improvement from the 28% probability of biventricular function at birth without intervention.^20^

Both the disease physiology and fetal heart intervention significantly alter the biomechanical environment of the fetal heart, which may influence its subsequent development. Studies in animal models have demonstrated that biomechanical aberrations can lead to cardiac maldevelopment and congenital heart malformations.^10^,^43^ The success with which minimally-invasive interventions improved structural heart development corroborated this idea, since such interventions are mechanical in nature. It is thus important to understand the biomechanics of fetal cardiac development and congenital heart malformations, such as critical AS, as this may improve our ability to predict gestational and perinatal outcomes.

To date, however, there have been few studies on the biomechanics of the fetal heart. Dewan et al. developed a finite element strain-based growth model for the fetal heart to test the hypothesis that restrictive mitral inflow could cause Hypoplastic Left Heart Syndrome (HLHS).^7^ Wiputra et al. performed computational modelling to delineate the fluid mechanics of structurally normal and abnormal fetal hearts with Tetralogy of Fallot.^51^ However, our understanding of the biomechanical environment of the fetal heart remains lacking. We address these knowledge gaps in the current study by performing image-based FE computational modelling of the biomechanics of the human fetal heart to understand the individual biomechanical effects of the various components of critical AS,

## Methods

Our strategy was to first develop FE models of the fetal LV myocardium based on clinical images of healthy fetal hearts, and subsequently modify these models to include various features of critical AS. Features investigated include AS, MR, LV hypertrophy, myocardial fibrosis, and cardiomyopathy.

### Data Acquisition and Processing

4D ultrasound images and valve Doppler velocities were acquired from cardiac imaging of three healthy fetuses at 22, 28 and 32 gestational weeks at the National University Hospital, Singapore. Procedures were approved by the Domain Specific Review Board under protocol 2014/00056, and informed consent was obtained from all participants. Retrospective Doppler velocity data and 2D B-mode images for an additional 32 fetal hearts with critical AS were acquired from the Kepler University Hospital (KUH), Austria. Procedures were approved under the KUH Institutional Review Board protocol #1009/2017.

**Figure 1:**
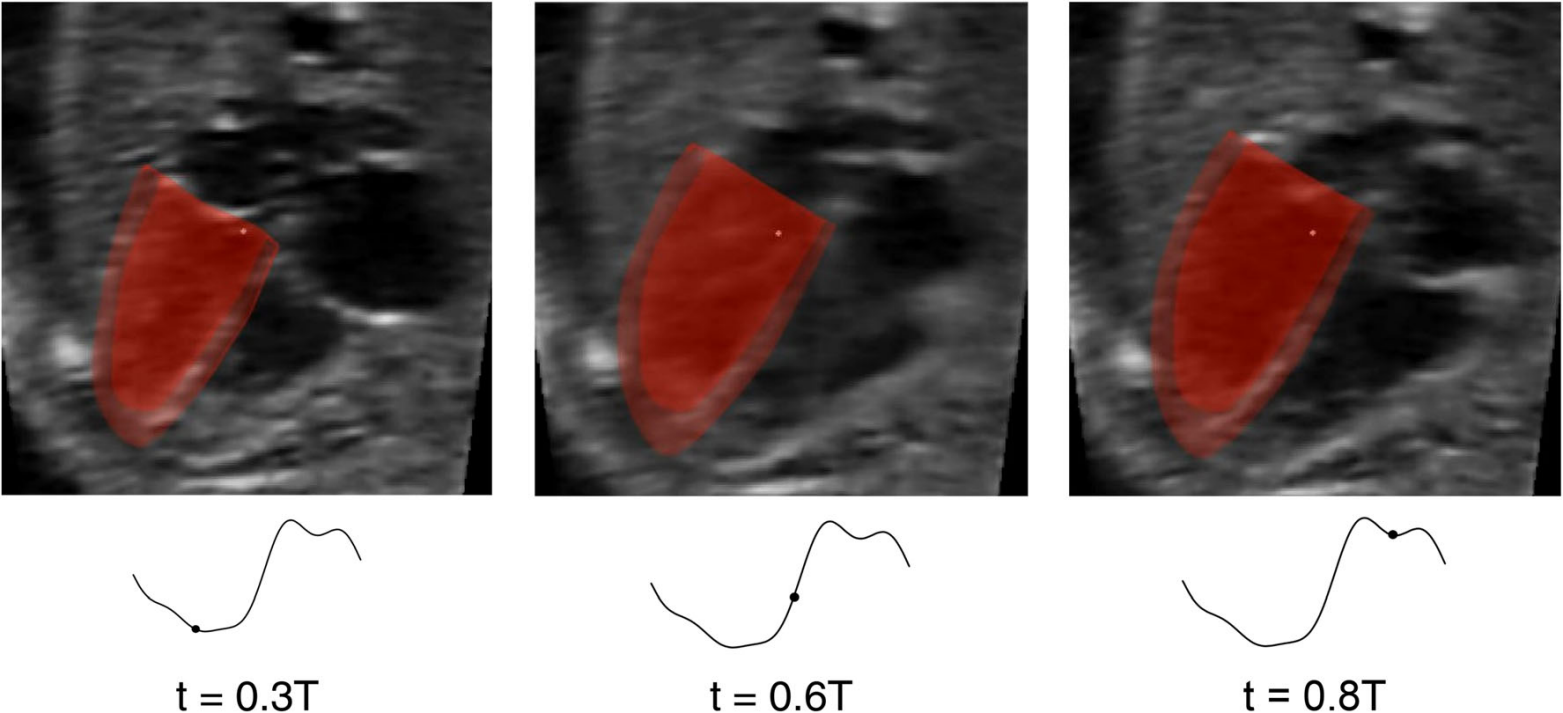
Three-dimensional fetal LV reconstructed from 4D ultrasound for a fetal heart at 22 weeks, with its motion tracked over the cardiac cycle, Here, t denotes time, while T denotes cardiac cycle duration.

4D B-mode images were acquired in the Spatio-Temporal Image Correlation (STIC) mode. Next, semi-automatic segmentation of the myocardium was performed with a custom-written lazy-snapping algorithm and vascular modelling toolkit (VMTK) software at a time point slightly before the end diastolic phase. The segmentation surface was then smoothed with Geomagic Studio® (Geomagic Inc., Morrisville, NC, USA), as previously described.^52^ A validated cardiac motion estimation algorithm was then applied to propagate the segmentation to other time points.^50^ This algorithm involved iterative curve-fitting of a global motion model (spatial B-splines of temporal Fourier function) onto a group of pair-wise image registration at various time points, and was validated *via* published echo data with MRI ground truths. Figure 1 and supplementary video 1 demonstrate proper tracking of myocardial wall motion.

### Mechanical Model of the Fetal Left Ventricle

The mechanical model and FE modelling of the fetal LV was adapted from previous work,^46^ where further details can be found. The FE model is explained briefly below and in the supplementary text. LV stress was decomposed into active and passive stress tensors. The passive stress tensor was modeled using a Fung-type transversely isotropic, hyperelastic constitutive model^21^ with the strain energy function, *W,* given by

1 $$W = \frac{1}{2}C\left( {e^{Q} - 1} \right),$$

where *C* is defined as passive stiffness coefficient with

2 $$Q = b_{ff} E_{ff}^{2} + b_{xx} \left( {E_{ss}^{2} + E_{nn}^{2} + E_{sn}^{2} + E_{ns}^{2} } \right) + b_{fx} \left( {E_{fn}^{2} + E_{nf}^{2} + E_{fs}^{2} + E_{sf}^{2} } \right)$$

In Eq. (2), *E*_***i****j*_ with (*i, j*) ϵ (*f, s, n*) are components of the Green-Lagrange strain tensor ***E***, with *f, s,* and *n* denoting the myocardial fiber, sheet and sheet normal directions, respectively.

Past studies had derived varied conclusions about the passive stiffness of the fetal ventricular myocardium. McPherson et al.^33^ concluded that there was no difference in stiffness between human fetal and adult myocardium, Friedman et al.^15^ found the fetal lamb myocardium to be stiffer than in the adult sheep, and Lahmers et al.^27^ reported similar findings in the porcine myocardium. We took a moderate position in light of this variability and assumed that fetal and adult myocardium had the same passive stiffness. Correspondingly, we adopted previously published adult myocardium passive properties^46^ for our fetal heart models.

The active stress, *P*_act_, was prescribed to act in the local fiber direction using a published active contraction model,^22^

3 $$P_{\text{act}} \; = \;T_{\hbox{max} } \frac{{Ca_{0}^{2} }}{{Ca_{0}^{2} + ECa_{50}^{2} }}C_{t} e_{f} \otimes e_{{f_{0} }}$$

where, *e*_*f*_ and *e*_*f*0_ are, respectively, the local vectors defining the muscle fiber direction in the current and reference configurations, *T*_max_ is the isometric tension as a result of the longest sarcomere length, and $Ca_{0}$ denotes the peak intracellular calcium concentration. $ECa_{50}$ is the length-dependent calcium sensitivity variable, and $C_{t}$ is a time-dependent variable.

Peak myocyte active tension, *T*_max_, was assumed to be 60 kPa, as an appropriate interpolation between early fetal and adult data. Racca et al. measured fetal myocardium active tension and found that fetal hearts at 130 days and 134 days could generate 49.9 ± 9.3 kPa and 36.0 ± 12.1 kPa, respectively.^42^ They found that adult hearts generated 75.2 ± 10.6 kPa, while Piroddi et al. measured this to be 108 ± 7 kPa.^41^ We thus believed that 60 kPa to be an appropriate estimate for *T*_max_ in our fetal heart models of all ages. This was corroborated by difficulty in matching the target stroke volume and pressures in our simulations of the 32 week old fetal heart when we had assumed a higher peak active tension of 80 kPa or 100 kPa. We did not assume a variability of *T*_max_ with gestational age due to the paucity of data. This simplified assumption is a limitation of the study.

Further details and values of parameters used are given in section S1 of the supplementary text.

### Finite Element Model Development and Calibration

**Figure 2:**
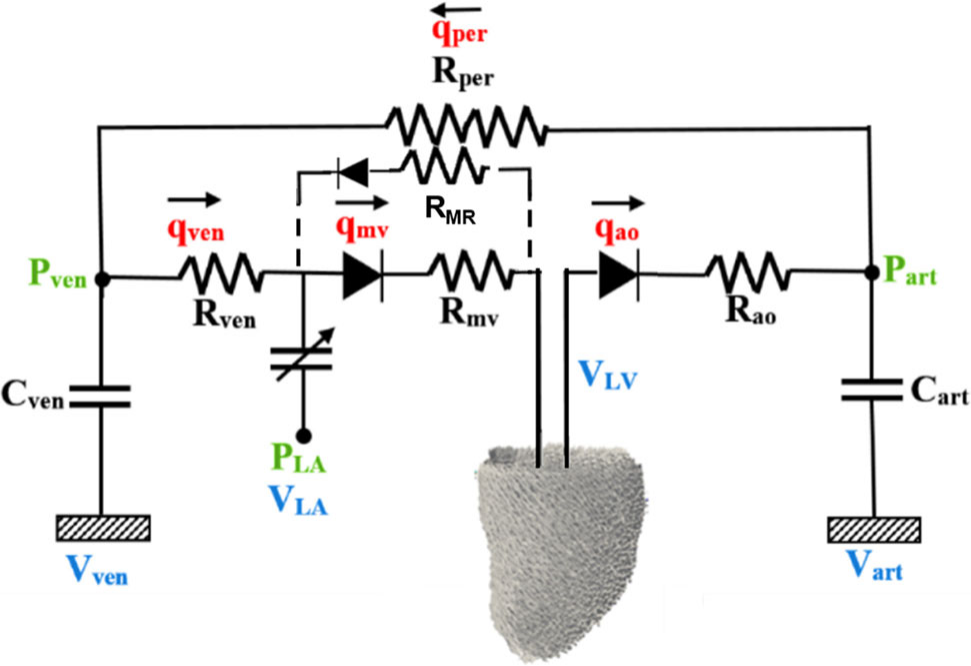
Windkessel model employed in the simulations. Elements on the dotted line were not used in healthy scenario modelling, but were added during disease modelling to simulate MR. C is capacitance, R is resistance,

FE modelling was performed using the open source library FEniCS^28^ (https://fenicsproject.org/). Segmented and smoothed LV models were discretized using quadratic tetrahedral elements, with at least 2500 elements, which was higher than past published work,^45^ and which passed our mesh convergence test of less than 2% change in the resulting stroke volume for a denser mesh size. The fetal LV base was constrained from moving out of the plane and the epicardial edge of the base was fixed.^49^ The myofiber helix angle was assumed to vary with a linear transmural variation from the epidcardium to the endocardium in the LV wall. A simplified Windkessel model, as shown in Fig. 2, was adopted to model the resistances of flow into and out of the LV but was not intended to model other details of the fetal circulation. The initial LV geometry was taken slightly before end-diastole. FE simulation was first performed to increase ventricular pressure to the end diastolic state, and then simulations were conducted for at least 10 additional cardiac cycles before results were analyzed. This was necessary to achieve a steady time-periodic pressure and volume waveforms.

During FE model development and calibration, we attempted to achieve models with reasonable outputs that matched characteristics obtained from the ultrasound images and from available literature. Peak LV systolic pressures were compared to literature values, ventricular volumes were compared to LV geometries obtained from the ultrasound images. The myofiber angle model and the Windkessel model were manually adjusted until a satisfactory match was achieved. This volume comparison was conducted at end-systole, when LV deformations were at their maximum difference from the end-diastolic starting geometry. The starting geometry was propagated to end-systolic state *via* ultrasound image tracking as described above and *via* FE simulations, and the resulting end-systolic geometry from both approaches were compared. Evaluation of the geometry match was conducted with the Iterative Closest Point algorithm in Geomagic Studio®,^26^ with 15000 random surface points and a tolerance of 0.001.

Healthy fetal LV pressure data were adopted from Johnson et al.^25^ who invasively measured this in human fetal hearts at gestational ages from 16 to 29 weeks, and provided an empirical linear equation for pressures versus age. We adopted this despite the need for extrapolation for our 32 weeks old fetus due to the lack of such data elsewhere in the literature.

The epi-to-endo myofiber angle that provided the best geometry match with ultrasound images were − 60° to 60° for the 32 weeks old fetal heart, –37° to 80° for the 28 weeks old heart, and –37° to 83° for the 22 weeks old heart, where 0^o^ denoted alignment to the circumferential axis, while 90^o^ denoted alignment to the apical-basal axis. Our endo-to-epi angles were close to 120^o^. These values were similar to literature values. Garcia-Canadilla et al. measured endo-to-epi angles of − 50^o^ to 100^o^ for mid-2nd trimester human fetus.^18^ Ohayon et al. measured approximately − 60^o^ to 60^o^ for 14, 20 and 33 weeks old fetal hearts,^40^ while Mekkaoui et al. reported no established fiber orientation for human fetal heart at 10 weeks, but by 19 weeks, there was a 120^o^ endo-to-epi angle difference.^34^

The Windkessel model parameters that enabled the good match are given in supplementary Table S2 and S3. The heart rate used in 22, 28, and 32 weeks old hearts were 147, 154 and 127 beats/min, which were obtained from their subject-specific ultrasound images, and which fitted within variability range in the literature.^47^

### Disease Features

Upon calibration of the healthy heart models, we adjusted parameters of to model the following disease features:

1. *Aortic stenosis.* To simulate this, we increased aortic flow resistance in the Windkessel model, and considered 3 levels of severity, a 3-fold, 10-fold, and 100-fold increase in aortic resistance, and denote these scenarios as “R_AO_*3”, “R_AO_*10”, and “R_AO_*100”, respectively
2. *Aortic stenosis and mitral regurgitation.* MR often accompanies fetal AS.^11^ To model this, additional Windkessel elements were added to the AS cases to allow for systolic MR flow (Fig. 2). Three severity levels were again modelled, with the MR flow resistance being 10^6^, 10^4^, and 10^3^ times that of the forward mitral inflow resistance as shown in Fig. 2 dotted line. We denote these scenarios as “R_MR_10^6^”, “R_MR_10^4^”, and “R_MR_10^3^”, respectively. The case of “R_AO_*100, R_MR_10^3^” demonstrated valve velocities results that were close to clinical observations in our critical AS cases. This model was used as the baseline for modelling disease features 3–5 below
3. *LV hypertrophy*. Increased wall thickness can accompany critical AS.^31^ To model this, the epicardial boundary of our LV geometry was offset outwards to generate thicker LV walls without reducing luminal volume. We investigated 10%, 25%, 50% and 100% increase in wall thickness
4. *Endocardial Fibroelastosis*. Endocardial fibroelastosis is commonly observed for fetal hearts with critical AS.^32^ We thus investigate the effects of myocardial stiffness changes, using scenarios of stiffness increased by 50% and 100%, *via* scaling of parameter C in Eq. (1)
5. *Cardiomyopathy*. Cardiac contractility can be weakened in some cases of AS.^24^ We investigated scenarios where the active tension was reduced, by reducing T_max_ in Eq. (3) to 66% and 50% of its original value

### Valve Flow Velocity Formulation

From the FE and Windkessel model, flow at the valves was modelled as volume flow rates. However, valve flow is typically quantified as Doppler velocities in clinical measurements, and volume flow rate is difficult to measure directly, due to difficulties in accurately measuring valve orifice size and acquiring good Proximal Iso-velocity Surface Area (PISA) images. We thus adopted an empirical formulation to convert volume flow rate to flow velocities to enable clinical interpretations of our results. Valve volume flow rate data was converted to flow velocities *via* the discharge coefficient ($C_{d}$) formulation. We adopted the empirical $C_{d}$ formula below,^12^ which was based on work by Nakayama et al.^38^*via* studies of jet flow through small orifices:

4 $$C_{d} \; = \;\frac{{\text{Re}^{{\frac{5}{6}}} }}{{17.11\frac{l}{d}\; + \;1.65\text{Re}^{0.8} }}$$

where $l$ and $d$ are the axial length and the radial diameter of the orifice respectively, and *Re* is the Reynolds number, calculated with the average velocity within the orifice, diameter of the orifice, and fluid viscosity. We adopted a kinematic viscosity of 4*10^−6^ (m^2^/s) from the literature.^4^ Nakayama et al.’s model was considered because it was formulated for *l/d* in the range of 1.5 to 17 and Re in the range 550 to 7000, and the conditions found in our study were within these ranges.

The thickness of the heart valves can be used to estimate the axial length of the orifice flow. We estimated the thickness of the aortic and mitral valves of fetal heart by first obtaining literature value of newborn heart valve leaflet thicknesses,^44^ and then scaling them down assuming a direct linear relationship with cardiac diameter.^19^ Thickness values used were 0.25 and 0.27 mm for the mitral and aortic valves. These thickness values were then assumed to be the axial length of the stenotic / regurgitant valve orifices.

*C*_*d*_ can be equated to pressures and flow rates *via* the Euler number (*Eu*),

5 $$Eu\; = \;\frac{1}{{C_{d}^{2} }}\; = \;\frac{\Delta P}{{\frac{1}{2}\rho u^{2} }}$$

where ∆P is pressure difference across the orifice, ρ is the blood density (1000 kg/m^3^),^36^*u* is average velocity within the orifice, which can be calculated from the volume flow rate and orifice size. Equation (5) was then solved with a simple gradient descent algorithm as follows (Eq. 6), and its results were manually verified.

6 $$\arg \mathop {\hbox{min} }\limits_{d} \left( {\frac{1}{2}\rho u^{2} - \Delta PC_{d}^{2} } \right)$$

## Results

**Figure 3:**
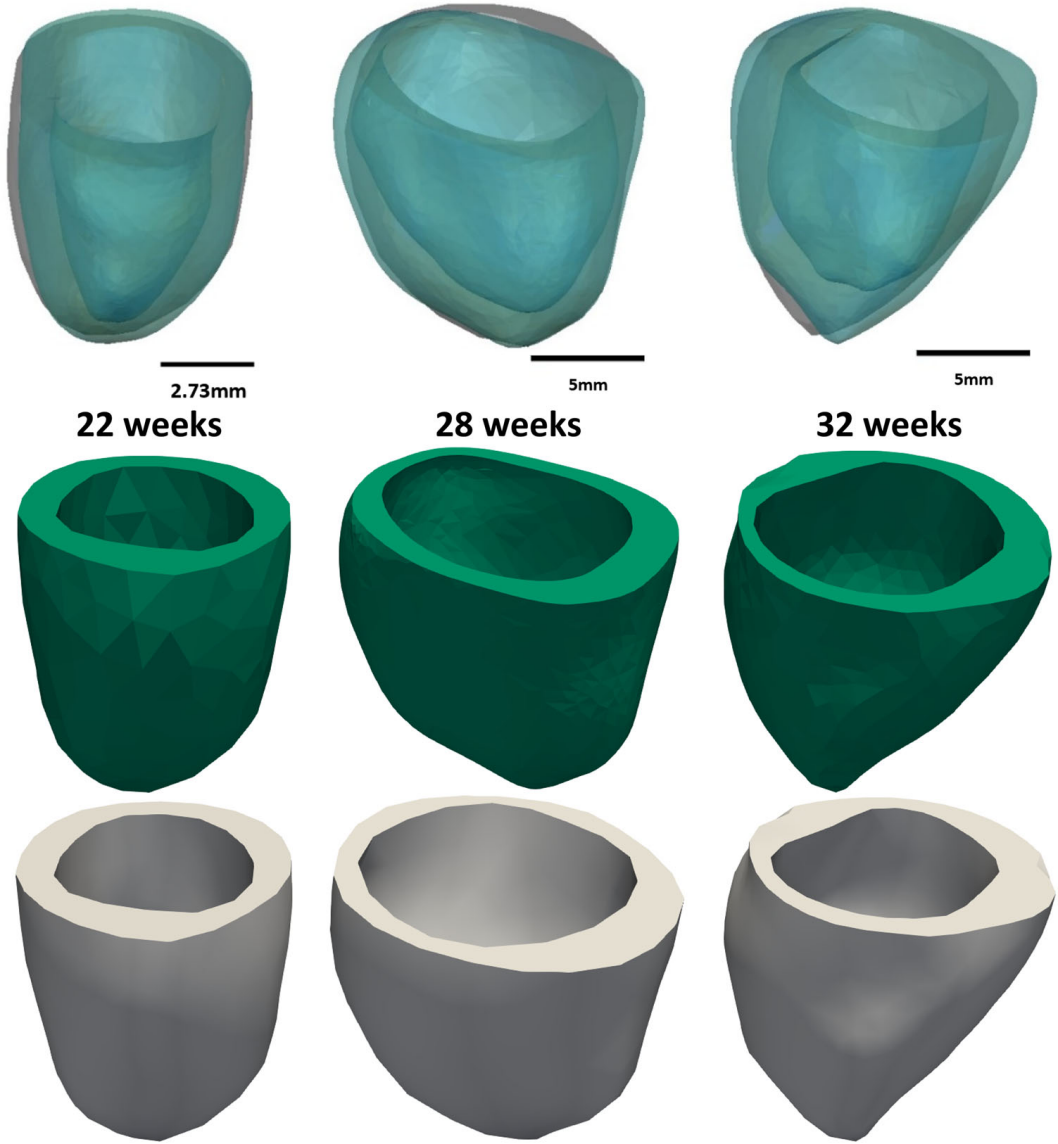
The end-systolic LV geometries of the healthy fetal hearts studied, obtained from FE modelling (green), and from ultrasound image motion tracking (gray). Geometries from both analyses showed a good match. The input to both FE modelling and motion tracking were the segmentation of the end-diastolic LV geometry.

The end-systolic myocardium geometry produced by FE modelling matched well with that produced by cardiac motion estimation from ultrasound images (Fig. 3). The average surface distance error was quantified to be less than 0.060 mm for all 3 hearts, which was less than 1% of the diameter of the smallest heart. The end-diastolic geometry from both analyses also matched well, but this was a trivial result since the end-diastolic geometry was the starting geometry for both analyses. A further quantification at 5 different time points spread out over the cardiac cycle (demonstrated in supplementary Fig. S4) showed that the distance error was were 0.038 ± 0.018 mm, 0.058 ± 0.033 mm, and 0.070 ± 0.030 mm of the respective for the 22, 28 and 32 weeks old hearts.

In addition, the LV volumes and stroke volume predicted by the models matched those obtained from ultrasound images (Table 1). Further, the stroke volume and chamber volumes of the 22 and 28 weeks old fetal hearts in this study were close to the mean values of their age-matched population reported in the literature, suggesting that they are suitable representation of their population. The 32 weeks old cases, however, was larger than the average of its age-matched population.

**Table 1:** Volumes and pressures of the FE LV normal fetal hearts studied, compared to quantifications from their 4D ultrasound images, and compared to literature values of their age-matching cohort

	Literature(*n* = 63)	Clinical Image	FE Model
*22* *weeks*			
End diastole volume (ml)	1.17 ± 0.28	0.94	0.89
End systole volume (ml)	0.60 ± 0.19	0.60	0.55
Stroke volume (ml)	0.54 ± 0.16	0.35	0.36
Pressure (mmHg)	27.0		30.12
*28* *weeks*			
End diastole volume (ml)	2.89 ± 0.68	2.05	2.15
End systole volume (ml)	1.61 ± 0.45	1.17	1.32
Stroke volume (ml)	1.30 ± 0.36	0.88	0.83
Pressure (mmHg)	37.2		32.87
*32* *weeks*			
End diastole volume (ml)	3.27 ± 0.86	5.22	5.45
End systole volume (ml)	1.95 ± 0.56	3.72	3.87
Stroke volume (ml)	1.41 ± 0.44	1.49	1.59
Pressure (mmHg)	44.2		40.2

End-diastole and end-systole volumes and stroke volumes (means ± SD) were obtained from Ref. 48, while pressures were calculated with an empirical equation from Ref. 25

### Effects of Aortic Stenosis and Mitral Regurgitation

**Figure 4:**
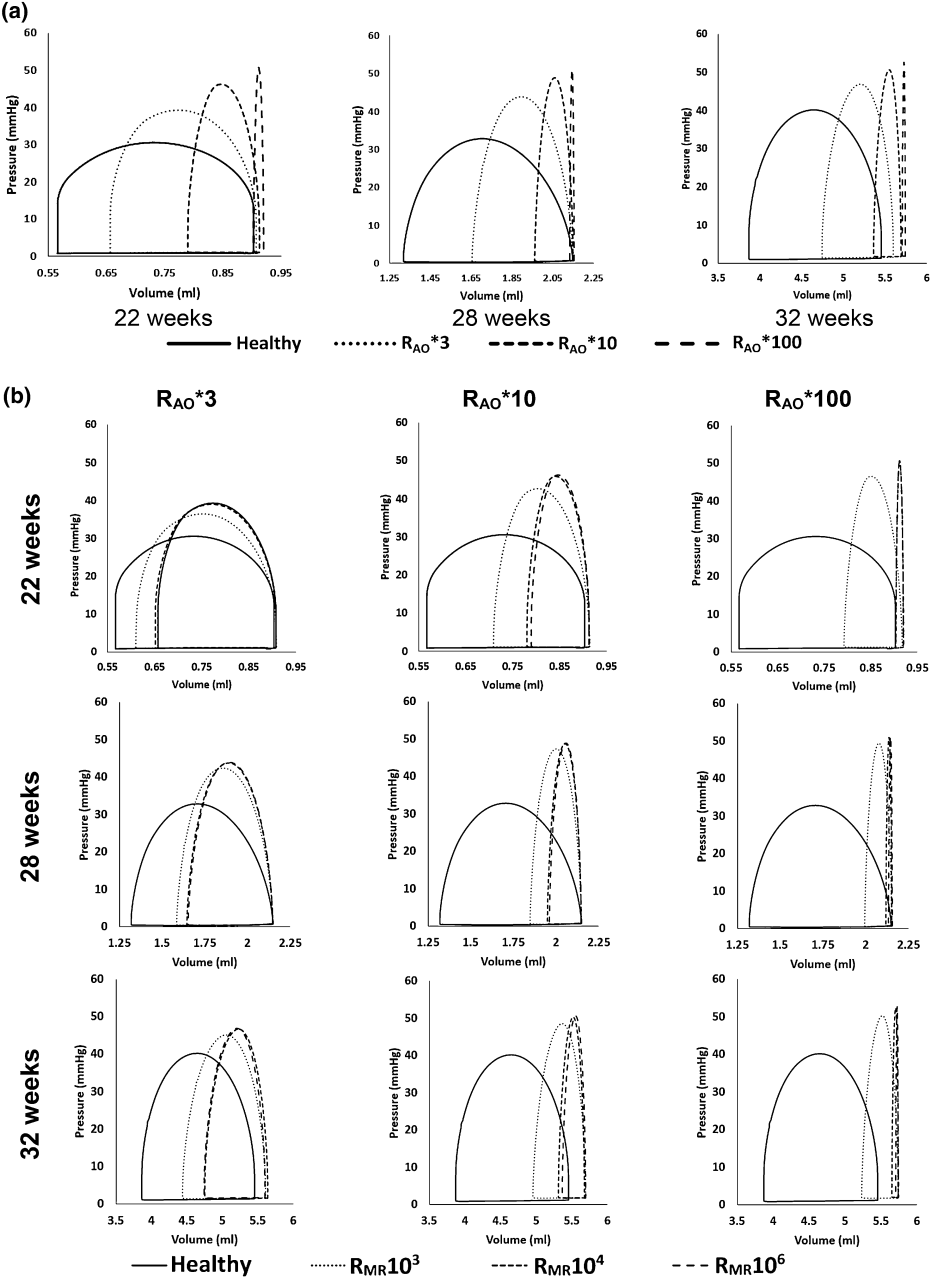
PV loop from FE simulations of healthy fetal hearts of various gestational ages, and the same hearts with various disease severity. (a) AS alone compared to healthy hearts. (b) AS with MR compared to healthy hearts.

Systolic pressure increased and stroke volume decreased with increasing AS severity, which was simulated by increasing the aortic valve resistance (Fig. 4a). The results showed that the R_AO_*100 was a sufficiently severe condition with stroke volume reducing to near zero. At this condition, LV pressures became elevated by about 10–20 mmHg above their healthy state. When MR was included to the AS cases, stroke volumes were partially restored and peak pressures were decreased (Fig. 4b). Generally, pressure reduction was modest (up to about 5 mmHg) for the range of mitral flow resistances explored.

**Figure 5:**
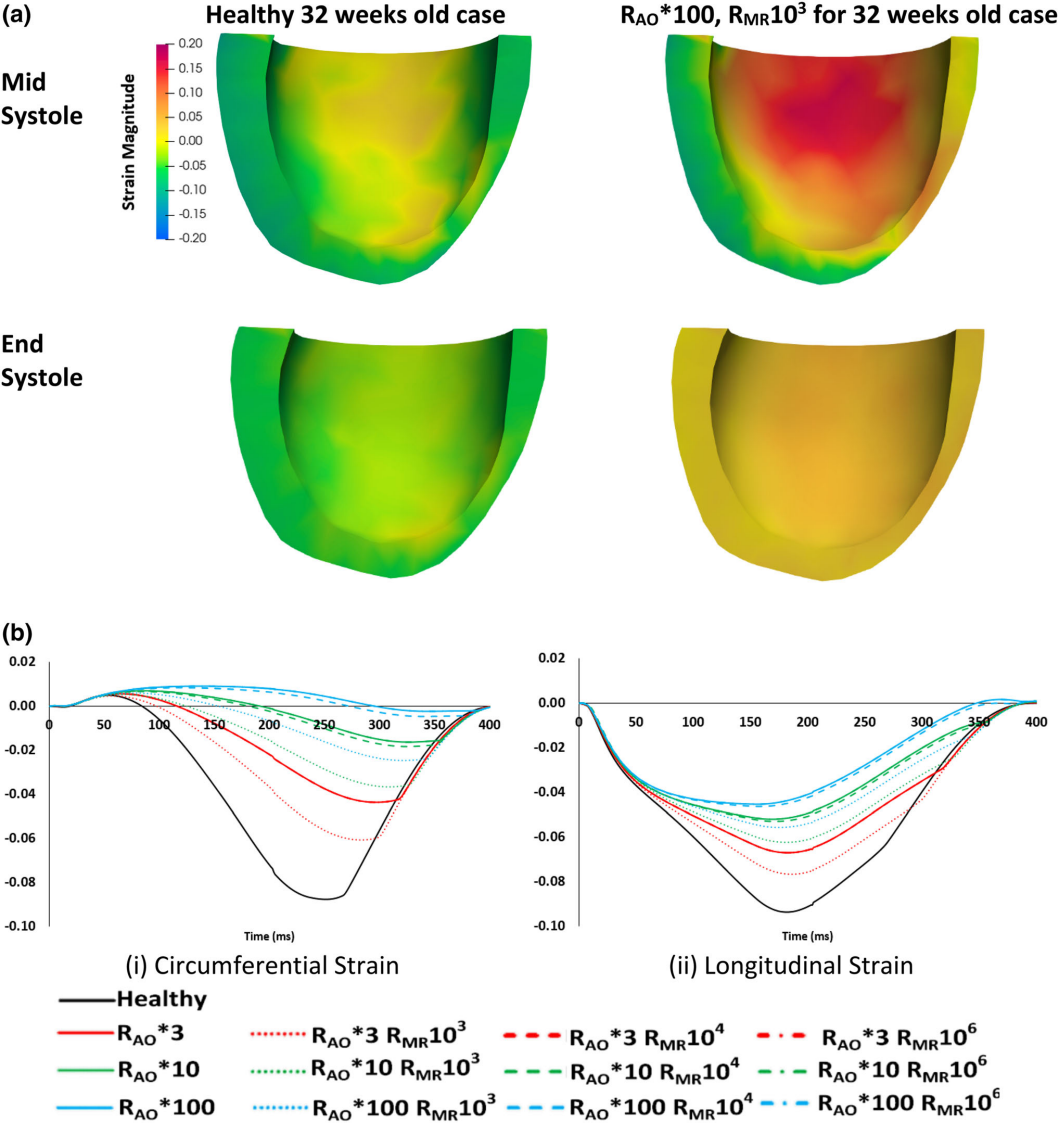
(a) Spatial pattern of fiber-direction strain for healthy and diseased (R_AO_*100, R_MR_10^3^) case for 32 weeks old fetal heart. (b) Spatially-averaged circumferential and longitudital strains over time of the 32 weeks old fetal heart, for the healthy and diseased (various severity) cases.

Figures 5a and 5b supplementary video 2 shows the spatial patterns of the fiber strain for the 32 weeks old fetal heart model in healthy and diseased (R_AO_*100 R_MR_10^3^) conditions. The spatial strain pattern was largely similar between the healthy and diseased cases, but the magnitude was different and reduced with AS. Figure 5b, which plots the temporal waveform of the spatially averaged longitudinal and circumferential strains for the 32 weeks old fetal heart model, demonstrates this further. With AS alone, both longitudinal and circumferential strains magnitude reduced with increasing severity of the AS. The decrease in circumferential strain magnitude was greater than that in longitudinal strain magnitude. Strain could be reduced to near zero value at R_AO_*100. With MR, this decrease in strain were less pronounced, which was similar to observations for stroke volume. Peak strains for all hearts are summarized in supplementary Fig. S1, and reflect largely the same trend as in Fig. 5.

**Figure 6:**
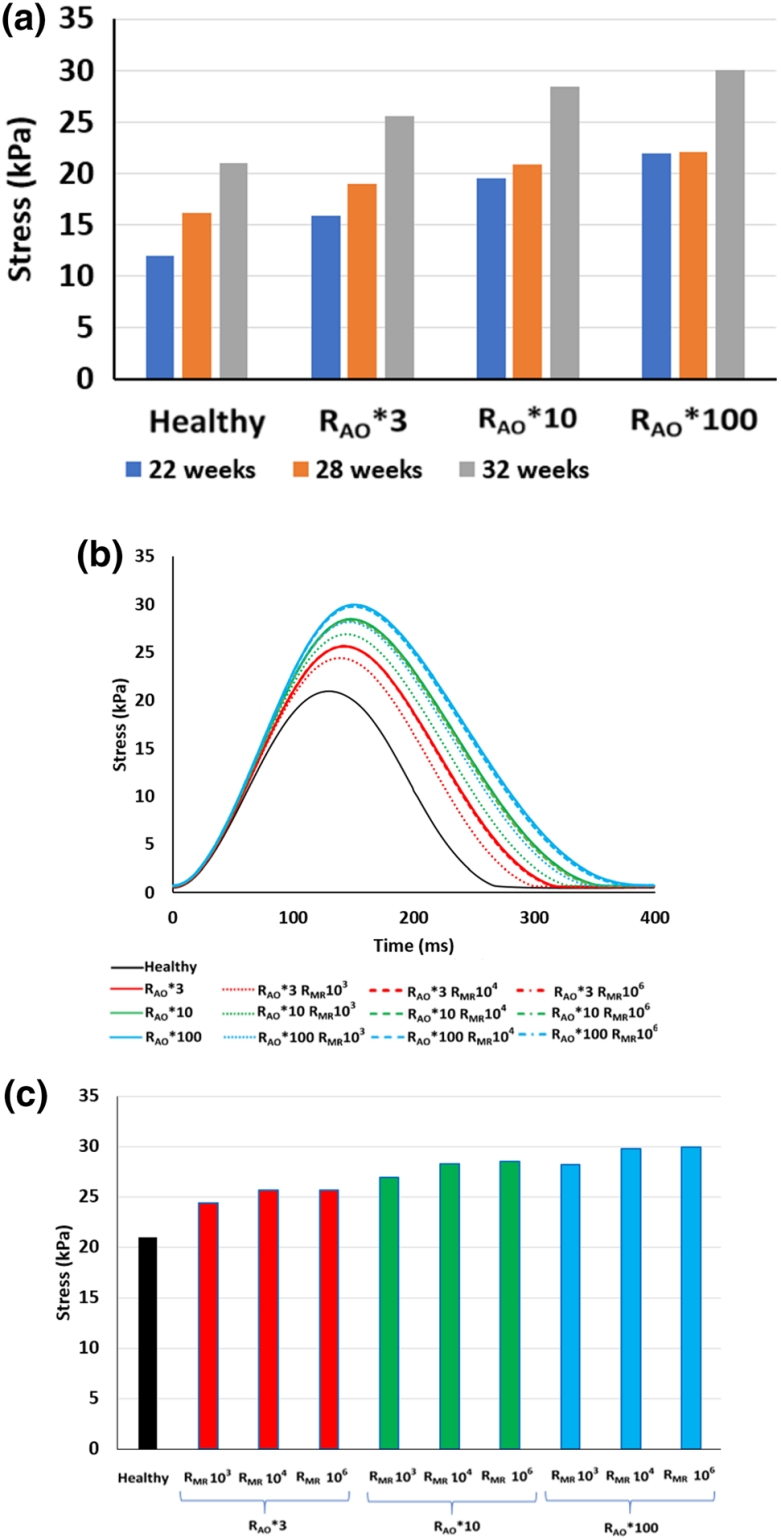
(a) Temporal peak of the spatial-averaged stress in the myofiber direction, for hearts of various gestational age, in health and during AS. (b) Spatially-averaged stress in the myofiber direction across time for the 32 weeks old fetal heart, comparing healthy and various disease scenarios. (c) Quantification of the temporal peak stresses from (b).

Stress in the myofiber direction generally increased with gestational age. From 22 to 32 weeks, this stress increased by 43.1% (Fig. 6a). This increase can be attributed to a 24% increase in systolic pressures, an 83% increase in LV luminal space, and a ~ 56.4% increase in wall thickness in the same period. Stress in the myofiber direction also increased with AS, with the increase approximately proportional with the elevations of LV systolic pressures (Fig. 6a). With MR, the stress decreased in the stenotic hearts (Figs. 6b and 6c). Figure 6b shows that the spatially-averaged stress waveform was similar to the LV pressure waveform. Similar data for the 22 and 28 weeks old fetal hearts are given in Figs. S2 and S3 in the supplementary text.

**Figure 7:**
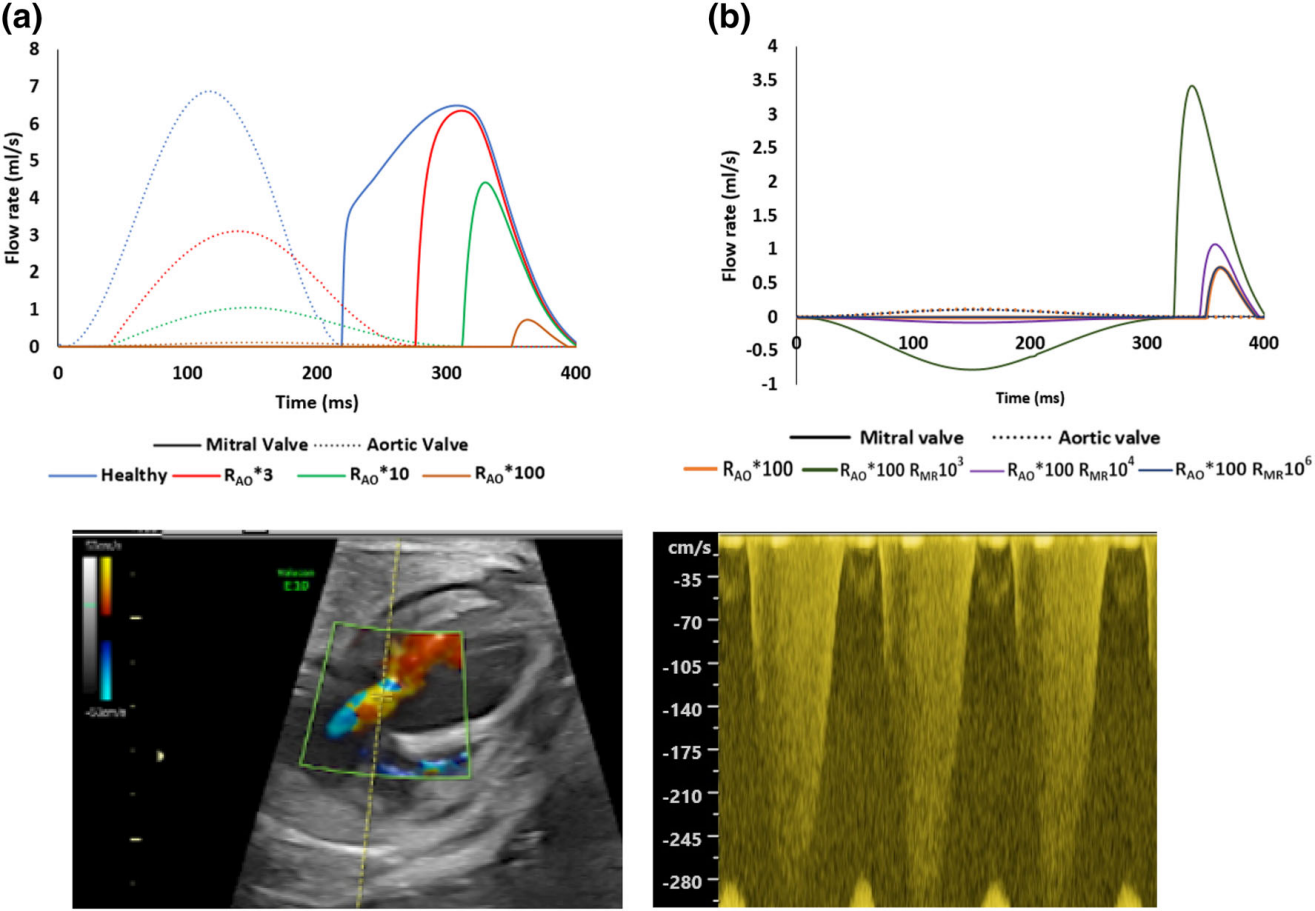
(a, b) Flow rates at the mitral and aortic valves under healthy and different disease condition for the 28 weeks old fetal heart. (a) Healthy case compared to cases with only AS. (b) The AS case of R_AO_*100 compared to the same case with various severity of MR. (c) Mitral Doppler velocity measurements in a 29 weeks old fetal heart with AS and MR, demonstrating long durations of regurgitation but short durations of mitral inflow.

Interestingly, with AS and MR, the systole-diastole timing was changed, as could be observed from the flow rate waveforms across the valve (Figs. 7a and 7b). A decrease in the mitral inflow diastolic duration and an increase in the aortic outflow duration was observed. With AS alone, diastolic duration could be reduced by up to 74% in the most severe simulation for 28 weeks heart, up to 68% for the 28 weeks heart, and up to 88% for the 32 weeks heart. When MR accompanied AS, this reduction was partially alleviated and diastolic duration was increased slightly. The same observation can be made from Doppler data of our KUH dataset, where systole-diastole duration ratios was found to be 3.16 ± 1.57, which meant 74 ± 10% of the cardiac cycle was systole, and 26 ± 10% was diastole. A sample Doppler image is shown in Fig. 7c.

The valve velocity estimated using the coefficient of discharge formulation is shown in Table 2 for the 28 weeks old fetal heart (also plotted in supplementary Fig. S5). Data for the other 2 hearts shows largely the same trends and are given in the supplementary Tables S4 and S5. Aortic outflow and MR velocities of the cohort of 32 critical AS fetal cases are also given for comparison. The aortic valve velocities from stenotic heart simulations were higher than literature values of aortic valve velocities for healthy hearts. Interestingly, we found that even when the AS was sufficiently severe to reduce stroke volume to close to zero, the aortic velocity could remain high, above 2 m/s. More severe AS was found necessary to decrease the aortic velocity further. This suggested that fetal LV stroke volume might be more sensitive to AS severity than aortic velocity. Our data also showed that at a milder stenosis, an increase in stenosis level (from R_AO_*3 to R_AO_*10) led to an increase in aortic velocity, but at a more severe stenosis, increasing stenosis (from R_AO_*10 to R_AO_*100) led to a decrease in aortic velocity.

**Table 2:** Peak flow velocities (m/s) at the valves of fetal hearts at various gestational ages, calculated based on discharge coefficient formulation for various diseased scenarios, compared to normal hearts from the literature;^2^ and the average velocities measured *via* Doppler ultrasound from our cohort of clinical AS and MR cases.

	22 weeks		28 weeks		32 weeks	
Healthy heart^2^	AV: 0.634 ± 0.225		AV: 0.848 ± 0.256		AV: 0.963 ± 0.277	
R_AO_*3	AV:	2.204	AV:	2.516	AV:	2.685
R_AO_*10	AV:	2.504	AV:	2.587	AV:	2.782
R_AO_*100	AV:	1.983	AV:	1.788	AV:	2.130

	R_AO_*3				R_AO_*10				R_AO_*100			
R_MR_10^3^	AV:	2.646	MV:	2.566	AV:	2.709	MV:	2.671	AV:	1.903	MV:	2.737
R_MR_10^4^	AV:	2.687	MV:	1.586	AV:	2.754	MV:	1.692	AV:	1.954	MV:	1.761
R_MR_10^6^	AV:	2.692	MV:	0.036	AV:	2.759	MV:	0.040	AV:	1.960	MV:	0.043
Average velocities in our cohort of 32 fetuses with critical AS and MR, with gestation age of 26.44 ± 3.37 weeks AV: 2.354 ± 0.783 m/s MV: 3.400 ± 0.854 m/s												

AV: aortic valve, MV: mitral valve

We further found that the aortic velocities in our simulated stenotic cases were close to the mean of the peak aortic Doppler velocities measured for our diseased fetal heart cohort (2.35 m/s). However, for peak MR velocities, our simulation results showed a range from close to 0 m/s to less than 3 m/s, which were lower than that clinically measured in our diseased cohort (3.40 m/s). Further manipulation of the MR resistance could not increase mitral velocities above 3 m/s, unless thickening of the LV wall was modelled, as described in analysis below. We thus noted that LV remodelling, such as hypertrophy, appeared to be necessary to increase MR velocities above 3 m/s.

### Effects of LV Hypertrophy

Manual quantification of the LV free wall thickness (3 measurements per echo image, 3 echo images per case) was conducted in 10 randomly selected cases from our KUH data set (average age = 28.0 ± 1.6 weeks). The free wall thickness was found to be 6.0 ± 1.1 mm, which is 105 ± 37% larger compared to value reported^29^ for age-matched healthy fetal hearts (2.9 ± 0.3 mm). The wall thicknesses of the 3 healthy fetal heart models used in our study were: 2.1, 2.9 and 3.3 mm.

**Figure 8:**
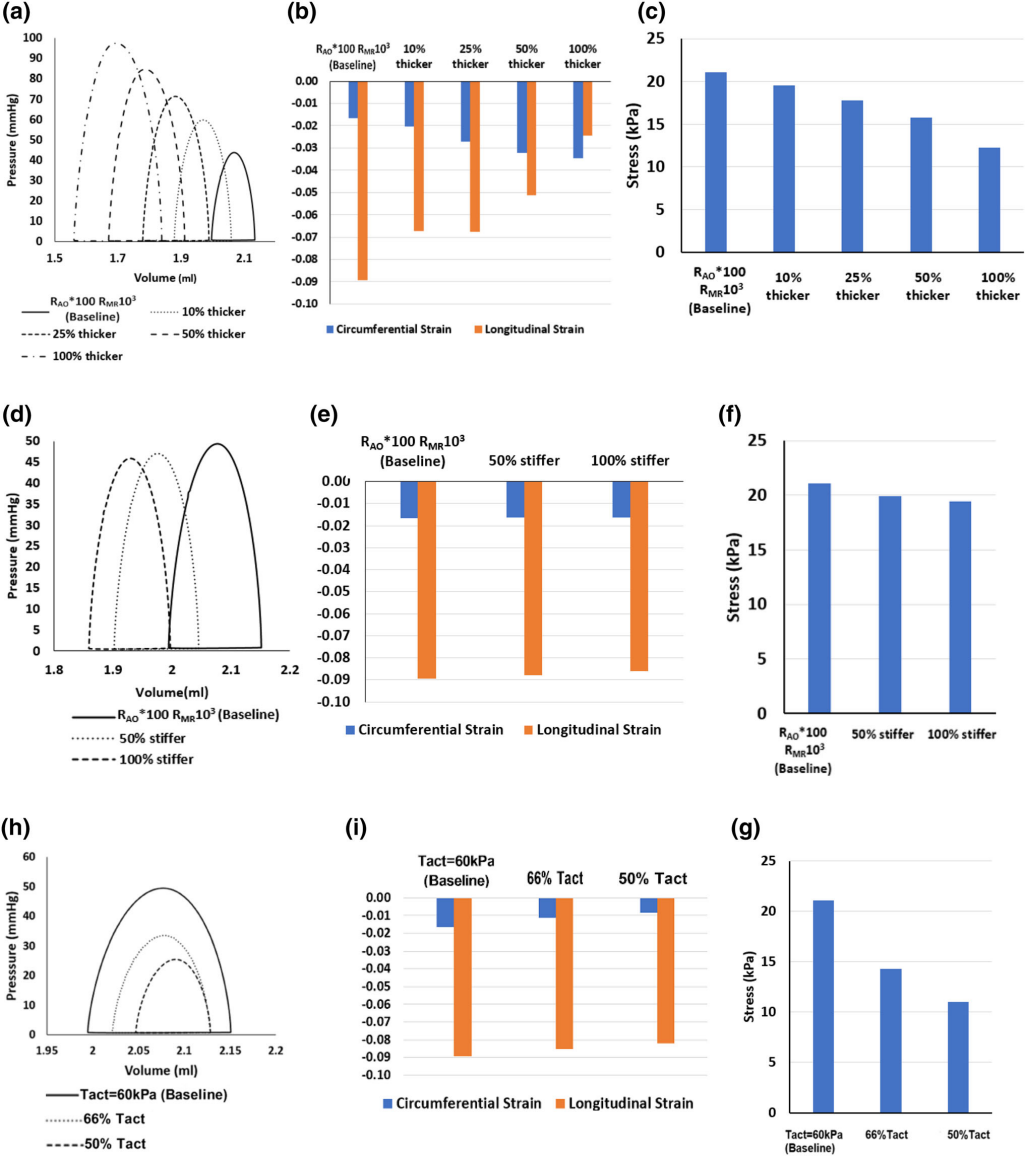
Effect of (a–c) LV wall thickening, (d–f) increasing LV stiffness, and (g–i) decreasing LV contractility on the (a, d, g) PV loop, (b, e, h) temporal-peak, spatial-averaged circumferential strain and longitudinal strain, and (c, f, i) temporal-peak, spatial-averaged myocardial stress in the fiber direction.

The results of simulations with increased LV wall thicknesses for the case of R_AO_*100, R_MR_10^3^ are shown in Figs. 8a–8c and Table 3A. With increasing wall thickness, stroke volume and LV systolic pressure increased, but myocardial stress in the fiber direction decreased. A 50% increase in thickness increased the peak systolic pressure by 72%, increased valve flow velocities from 2.8 m/s to 3.9 m/s, increased stroke volume by 54%, increased LV wall strain in the fiber direction by 35%, but decreased myocardial stress in the fiber direction by 25%. In the 100% hypertrophy simulation case, however, the LV systolic pressure was 97.5 mmHg, higher than average value of approximately 63 mmHg reported for fetal AS cases.^53^ The valve velocities (Table 3A) were much higher than the average in our KUH dataset (Table 2), even though 100% hypertrophy was found to be the mean level of hypertrophy in our AS cases. This suggested that reduced contractility is likely to have accompanied AS cases with hypertrophy.

**Table 3:** Peak flow velocities (m/s) for (A) various wall thickness cases, (B) various wall stiffness cases, and (C) various LV contractility cases, modelled from the R_AO_*100, R_MR_10^3^ baseline condition

Case	Velocity (m/s)			
*(A) Effects of Increasing LV wall thickness*				
R_AO_*100, R_MR_10^3^ (baseline)	AV:	1.903	MV:	2.737
10% wall thickness increase	AV:	2.262	MV:	3.076
25% wall thickness increase	AV:	2.680	MV:	3.465
50% wall thickness increase	AV:	3.179	MV:	3.881
100% wall thickness increase	AV:	3.544	MV:	4.260
*(B) Effects of Increasing LV wall stiffness*				
R_AO_*100, R_MR_10^3^ (baseline)	AV:	1.903	MV:	2.737
50% stiffness increase	AV:	1.872	MV:	2.707
100% stiffness increase	AV:	1.884	MV:	2.718
*(C) Effects of decreasing LV contractility*				
T_max_ = 60 kPa	AV:	1.903	MV:	2.737
33% decrease in T_max_ (40 kPa)	AV:	1.337	MV:	2.176
50% decrease in T_max_ (30 kPa)	AV:	1.021	MV:	1.841

AV: aortic valve, MV: mitral valve

It was noteworthy that, after simulations of multiple cardiac cycles, thicker wall simulation cases demonstrated a reduction of the overall LV lumen size compared to the baseline case before thickening. This occurs even though we have modelled thickening only in the epicardial direction as the initial condition. This is because thicker walls reduced LV compliance,^35^ which slightly inhibited diastolic distention.

### Effects of Fibroelastosis

We used stiffness changes as a model for fibroelastosis. Results are shown in Figs. 8d–8f and Table 3B. Surprisingly, a substantial 50% increase in myocardial tissue stiffness produces only modest reduction of the peak systolic pressure (4.4%), ventricular size (6.1%), stroke volume (6.8%), and stress in myofiber direction (5.3%). Table 3B shows that aortic outflow and MR velocities were only mildly reduced.

### Effects of Cardiomyopathy

Results of modelling with decreased LV contractility are shown in Figs. 8g–8i and Table 3C. Unsurprisingly, the reduction produced a roughly proportional decrease in stroke volume and systolic pressure. Corresponding decrease in stress (myofiber direction), strains, reduced aortic outflow and MR velocities were also observed.

## Discussion

In this study, we developed and calibrated FE models of healthy fetal hearts from 4D ultrasound images and Doppler velocity measurements, and then used them to investigate biomechanical changes during disease conditions associated with fetal AS. We could successfully calibrate the FE models, where healthy heart models showed stroke volume, chamber sizes, and LV geometry that agreed well with the same data obtained from clinical images. When we used the calibrated FE model to investigate diseased scenarios, we could capture systole-diastole timing alterations observed in clinical measurements in disease cases, and our simulated valve velocity results were close to these measurements. The observation of alteration of systole-diastole timing under critical aortic stenosis cases was also reported in the literature.^8^ These good agreements between the simulation results and clinical measurements suggested that the FE models may be able to predict fetal heart biomechanics well, and may be a useful tool to understand normal and diseased heart physiology. FE modelling is further useful as it can estimate parameters that are difficult to measure *in vivo*, such as myocardial stresses.

The focus of our current work, however, was to investigate the individual effects of each of the disease features. For this reason, we did not study the interdependencies of the various features, and we used the healthy heart as a baseline for our investigation, instead of directly using the diseased heart anatomy and diseased myocardial characteristics as the baseline. This is also due to the lack of information of the diseased fetal myocardium. These are limitations of the current work that warrants future studies. However, the advantage of our chosen focus is that we can better understand the effects of each individual disease feature, which will be difficult with clinical or animal studies. Simulations results from the healthy baseline can also be helpful when considering the next question of how abnormal biomechanics can lead to pathological remodelling.

From our results, we found that AS can elevate fetal LV peak systolic pressures significantly (10–20 mmHg), but MR can reduce this slightly. With these two disease features, we observed that aortic velocity could be elevated to 2–3 m/s, which was similar to that in our cohort of fetal hearts with critical AS. However, interestingly, the MR velocity could not be elevated above 3 m/s, while many cases in our cohort of fetuses with critical AS had velocities of above 3.5 m/s. We found that LV wall thickening was necessary to increase LV pressures further for MR velocities to be elevated to this level, suggesting that such clinical cases might have hypertrophy.

Our simulations further showed AS decreases stroke volume and LV strains more readily than it decreased aortic flow velocity. In our most severe AS cases, stroke volume and strains were close to zero, but aortic velocities remained above 2 m/s. From our investigation, we found that further reduction of the aortic velocity was possible with further increase in the aortic resistance, and our simulations could produce aortic velocity that is close to zero if resistance was sufficiently high. From our clinical data samples, there were cases where the aortic velocity were less than 1 m/s, much lower than the average of the whole cohort.

Another noteworthy finding was that AS caused LV strain to decrease much more in the circumferential direction than in the longitudinal direction. Consequently, while our normal hearts had longitudinal strains that were close to their circumferential strain, which was in agreement with a previous study,^23^ our models predicted that diseased hearts had different longitudinal and circumferential strains. Fetal myocardium speckle tracking strain measurements from the literature, however, did not detect such a disparity.^24^ We propose that this mismatch between our simulations and clinical observations can be explained by stenotic fetal heart remodelling its myofiber orientations in a way that reduces the disparity between longitudinal and circumferential strains. We checked the feasibility of this notion with simulations that altered the fiber orientation and found that we could obtain a wide range of differences between longitudinal and circumferential strain. By manipulating fiber angles, strain in a particular direction could switch from positive (stretch) to negative (contract) values (supplementary Fig. S6).

Further, our simulations showed that they could capture the increase in the systole duration and decrease in the diastole duration observed in clinical AS cases. This was observed even without modulating the myocardial activation timing in our model. This phenomenon could be explained by the higher pressure in the LV associated with the disease, which required more time to decrease (*via* myocardium deformations) to a sufficiently low pressure for mitral inflow to occur.

There are two types of hypertrophy which is possible in the fetal heart, eccentric and concentric hypertrophy. Concentric hypertrophy is typically found in pressure overload situations, such as during fetal AS, while eccentric hypertrophy can be found in fetal growth restriction cases.^5^ In our study, we have modelled only concentric hypertrophy. An increase in LV wall thickness was found to have a significant effect on the hemodynamics and LV function. Changes in the wall thickness substantially enhanced the LV pressure, decreased the stress (myofiber direction) and increased stroke volume. This result is clinically relevant, since hypertrophy was observed in clinical cases of fetal AS,^31^ animal model of aortic banding,^10^ and the KUH clinical data set. It is unclear if this wall thickening is due to cell proliferation or cell enlargement, given that the fetal heart remodels differently from the adult heart.^13^ Our results, however, show that at the average level of hypertrophy of approximately 100% increase in wall thickness, LV pressures and valve velocities were much higher the average of these parameters in fetal AS patients, suggesting that reduced myocardial contractility is likely to have accompanied hypertrophied AS fetal hearts.

Reduced LV contractility substantially was observed to decrease LV pressure, strain and stroke volume. Contractility may be an important factor influencing the outcomes of fetal aortic valvuloplasty intervention in critical AS cases. Ishii et al.^24^ reported that fetal hearts with AS that demonstrated stronger contractions after aortic valvuloplasty tended to benefit from the intervention and achieve biventricular cardiac function at birth. Conversely, cases that demonstrated lower contractions commonly ended up with univentricular function at birth. With our image-based FE modeling approach, a potential future application is computational estimation of cardiac contractility for prognostication and to predict intervention outcomes.

Interestingly, we found that an increase in myocardial passive stiffness only had very modest effects on myocardial strains and stresses, stroke volume and pressure. This result suggests that endocardial fibroelastosis would not have a major effect on the cardiac function and might not be causing adverse outcomes. Past investigations, however, showed that the severity of fetal LV endocardial fibroelastosis were correlated to the abnormal LV globular geometry, reduced LV function, and lower probability of a biventricular birth outcome.^32^ We suggest that fibroelastosis may simply be a by-product of the disease, rather than being a factor impeding cardiac function to cause poor outcomes.

Compared to adult AS, fetal AS has some similarities and differences. In both scenarios, there are elevated LV pressures, which leads to hypertrophy.^3^,^6^ However, endocardial fibroelastosis is observed only in fetal AS,^32^ but not in adult AS. Further, the fetal heart undergoes remodeling and morphological changes, and can potentially develop into HLHS,^30^ but the adult heart only hypertrophies. In the fetal heart, it could further be possible for stenosis to be extremely severe, since the right ventricle can sustain circulation. Such a scenario will be fatal in adults, however. Much work has been done for adult FE modelling for pressure overloaded hearts,^37^ including modelling of growth and remodelling.^39^ Such work can be very useful if applied to the fetal heart, potentially predicting morphological changes subsequent to disease biomechanics.

There are some limitations associated with this work. Firstly, the small sample size of fetal heart cases used for simulations is a limitation. Secondly, our model omitted a description of the right ventricle, which might affect the spatial distribution of stresses. Thirdly, there could be many concurrent changes during fetal AS development, such as fiber orientation remodelling and changes to myocardial active tension, which were not considered here. Fourthly, our Windkessel model was a simplified representation that did not fully capture fine details of the fetal circulation, and further work to develop a more specific model is underway. Despite these limitations, we believe that the FE model is still useful in developing an understanding of the biomechanical effects of various disease features, and the general trends of these results are unlikely to be affected by these limitations. Finally, our process of optimizing parameters that enables a good geometric fit between the FE model and segmentations from images was conducted manually, and an automated algorithm is likely to further improve the fit.

In conclusion, we conducted a clinical image-based FE modelling of the human fetal heart and demonstrated that it can successfully model normal heart biomechanics as well as the effects of various disease features, such as AS, MR, LV hypertrophy, reduced contractility, and LV fibrosis. AS was found to elevate LV pressures and stress (myofiber direction), and reduce myocardial strains and stroke volume. MR moderates these effects. LV hypertrophy significantly increased LV pressures, strains, and stroke volume and was found necessary to produce high MR valve velocities as measured in the clinic. Reduced contractility significantly decreased LV function, but increased passive myocardial stiffness had only modest effect on LV function and its biomechanical environment.

## Electronic supplementary material

Below is the link to the electronic supplementary material.

Supplementary material 1 (DOCX 3544 kb)

Supplementary material 2 (MP4 1161 kb)

Supplementary material 3 (MP4 8235 kb)
