# Supplementary material for: Biomechanics of Human Fetal Hearts with Critical Aortic Stenosis
Source: Ann Biomed Eng. 2020 Nov 11;49(5):1364–79. doi: 10.1007/s10439-020-02683-x (PMC8058006; doi:10.1007/s10439-020-02683-x)
Supplement: Supplementary file 1 — Supplementary material 1 (DOCX 3544 kb) [file 10439_2020_2683_MOESM1_ESM.docx]

**Supplementary Method**

***S1. Mechanical Model of the Fetal Lett Ventricle***

Following section 2.2, the active contraction model we used is described here. $ECa_{50}$ is the length-dependent calcium sensitivity variable

${ECa}_{50}=\frac{\left( {Ca}_{0} \right)_{max}}{\sqrt{\exp\left( B\left( l-l_{0} \right) \right)-1}},$ (S1)

$C_{t}=\frac{1}{2}(1-cos\omega)$ (S2)

Where *B* is a constant, (*Ca_0_*) *_max_* is the maximum peak intracellular calcium concentration, $l$ is sarcomere length, and $l_{0}$ is the initial sarcomere length at which no active tension develops. The variable *ω* in Equation (S2) is given by

$\omega=\left\{ \begin{aligned} \pi\frac{t}{t_{0}}, 0\leq t<t_{0}; \\ \pi\frac{t-t_{0}+t_{r}}{t_{r}}, t_{0}\leq t<t_{0}+t_{r}; \\ 0, t_{0}+t_{r}\leq t \end{aligned} \right.$ (S3)

Where *t_0_* is the time taken to reach peak tension and *t_r_* is the duration of relaxation that depends linearly on the sarcomere length *l* by

$t_{r}=ml+B,$ (S4)

where m and b are constants. The sarcomere length *l* can be calculated from the myofiber stretch *λ_LV_* by

| $\lambda_{LV}=\sqrt{e_{f_{0}\cdot}\cdot C_{LV}e_{f_{0}}},$  $l=\lambda_{LV}l_{r}$ | (S5) |
| --- | --- |

In Equation (S5), ***C_LV_*** = ***F_LV_^T^F_LV_*** is the right Cauchy Green deformation tensor and *l_r_* is the relaxed sarcomere length. Parameter values linked to the LV model are listed in Table S1.

***Table S1.*** *Parameters of LV FEM model*

| Parameter | Description | Value |
| --- | --- | --- |
| *C* | material parameter, kPa | 0.1 |
| *b_ff_* | material parameter | 29.9 |
| *b_xx_* | material parameter | 13.3 |
| *b_fx_* | material parameter | 26.6 |
| *T_max_* | isometric tension under maximal activation, kPa | 60 |
| *C_a0_* | peak intracellular calcium concentration, µM | 4.35 |
| *(Ca_0_) _max_* | maximum peak intracellular calcium concentration, µM | 4.35 |
| *B* | governs shape of peak isometric tension-sarcomere length relation, µm-1 | 4.75 |
| l_0_ | sarcomere length at which no active tension develops, µm | 1.58 |
| t_0_ | time to peak tension, ms | 150.5 |
| *m* | slope of linear relaxation duration-sarcomere length relation, ms µm-1 | 524 |
| b | time-intercept of linear relaxation duration-sarcomere length relation, ms | 800 |
| l_r_ | relaxed sarcomere length, µm | 1.85 |

***S2. Windkessel Model Parameters***

Windkessel modelling is an established low dimensional physics modeling of hemodynamics.^2^ To determine Windkessel model parameters Our approach was as follows:

1. Determine the myocardial active tension force, material stiffness according to literature evidence.
2. As the initial guess, adopt model given by Shavik et al. for the adult heart, assumed fiber direction used by previous literature.
3. Manually adjust each Windkessel parameter and the two fiber angle parameters, one at a time, with 10%, 25% or 50% perturbations. Note errors in peak systolic pressure, stroke volume, and average surface error between simulated and image-segmented myocardium. Adopt the more accurate results.
4. Adopt results when average surface distance error was less than 1% and stroke volume and pressure errors are less than 7%

Following section 2.3, the Windkessel model parameters used are described here in table S2. The total blood volume used was between 112-117 ml for the 3 fetal cases.

***Table S2****. Resistance and capacitance value for the circulatory model*

|  | Units | 22 weeks | 28 weeks | 32weeks |
| --- | --- | --- | --- | --- |
| Aortic valve resistance, R_ao_ | Pa*msec*mL^-1^ | 704000.00 | 526120.00 | 332571.30 |
| Peripheral resistance, R_per_ | Pa*msec*mL^-1^ | 1774500.00 | 10360.44 | 209569.50 |
| Venous resistance, R_ven_ | Pa*msec*mL^-1^ | 500.00 | 186.83 | 118.10 |
| Mitral valve resistance R_mv_ | Pa*msec*mL^-1^ | 10000.00 | 7473.30 | 2362.01 |
| Aortic compliance, C_ao_ | mLPa | 6.50x10^-4^ | 2.41x10^-4^ | 4.43 x10^-3^ |
| Venous compliance, C_ven_ | mLPa | 0.02 | 0.04 | 1.70x10^-2^ |

**S3. Time varying elastance for the left atrium**

The time varying elastance model for the left atria in the Windkessel model was modelled according to previous literature.^1^ Briefly, the atrial pressure $P_{LA}\left( t \right)$ and the atrial volume $V_{LA}\left( t \right)$ are modelled as follows, and the parameters in the model are given in Table S3.

| $P_{LA}\left( t \right)=e\left( t \right)E_{es,LA}\left( V_{LA}-V_{0,LA} \right)+(1-e\left( t \right)A_{LA}(e^{B_{LA}\left( V_{LA}-V_{0,LA} \right)}-1)$ | (S6) |
| --- | --- |

Where

| $e\left( t \right)=\left\{ \begin{matrix} \frac{1}{2}\left( sin\left[ \left( \frac{\pi}{T_{max}} \right)t-\frac{\pi}{2} \right] \right); 0<t\leq3/2T_{max} \\ \frac{1}{2}e^{-(t-\frac{3}{2T_{max}})/\tau}; t>3/2T_{max} \end{matrix} \right.$ | (S7) |
| --- | --- |

***Table S3.*** *Parameters of time varying elastance model for left atrium*

| Parameter | Description | Value |
| --- | --- | --- |
| *E_es,La_* | End-systolic elastance, Pa/mL | 60 |
| *V_0,LA_* | Volume axis intercept, mL | 0.25 |
| *A_LA_* | Scaling factor for EDPVA Pa | 580.5 |
| *B_LA_* | Exponent for EDPVR, mL-1 | 0.049 |
| *T_max_* | Time to end systole, msec | 125 |
| $\tau$ | Time constant of relaxation, msec | 25 |

**Supplementary Results**


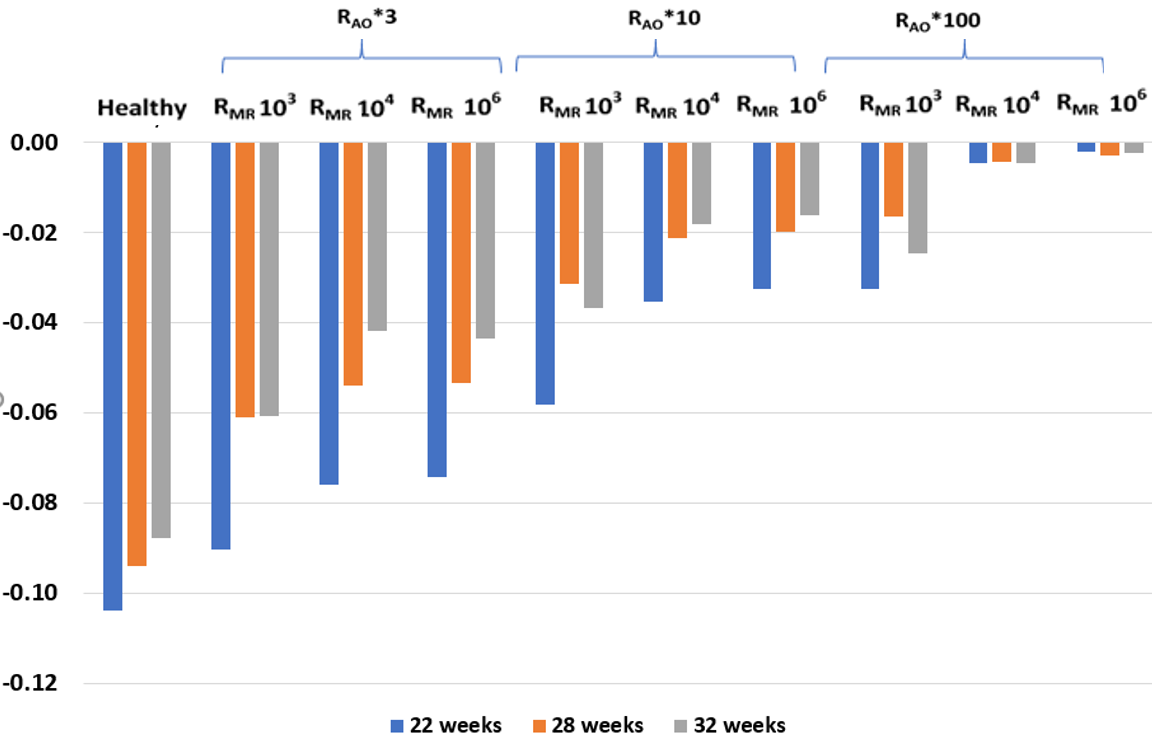


(A)


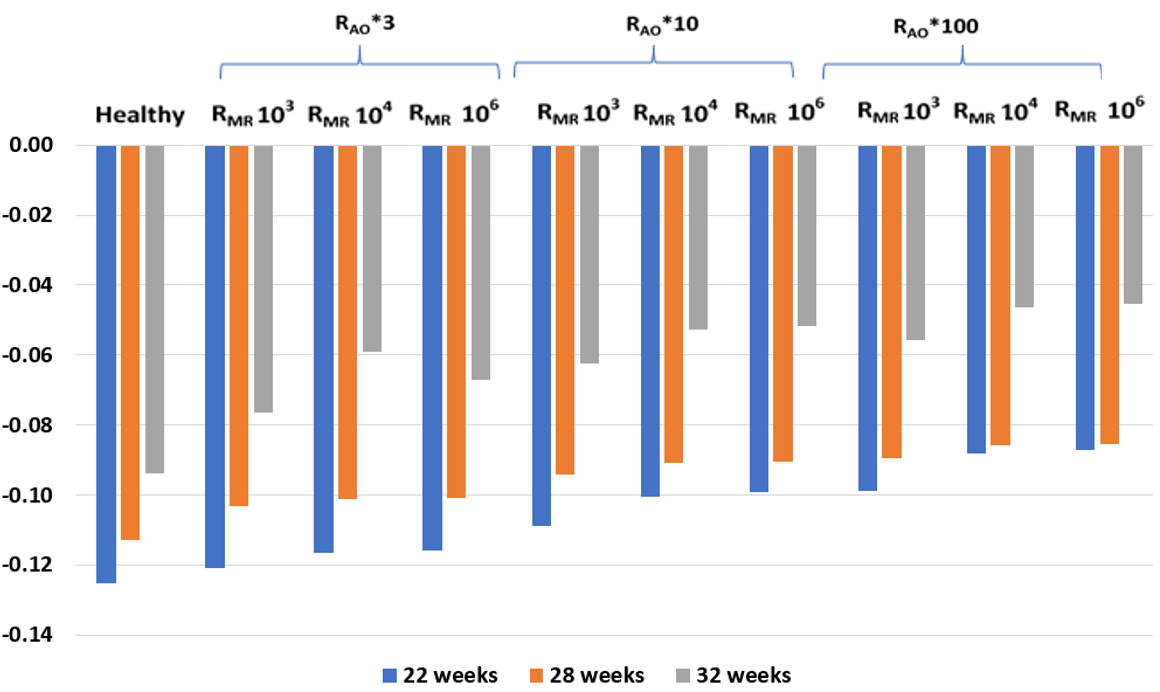


(B)

***Figure S1.*** *(A) Peak Circumferential Strain (B) Peak Longitudinal Strain obtained from FEM simulations of fetal hearts at various gestational ages, with aortic stenosis and mitral regurgitation of various severity.*


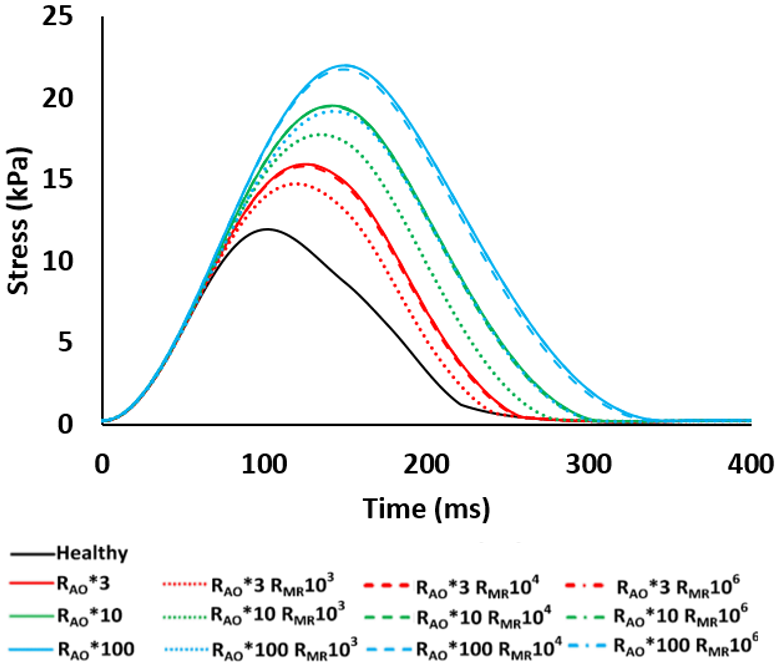


(A)


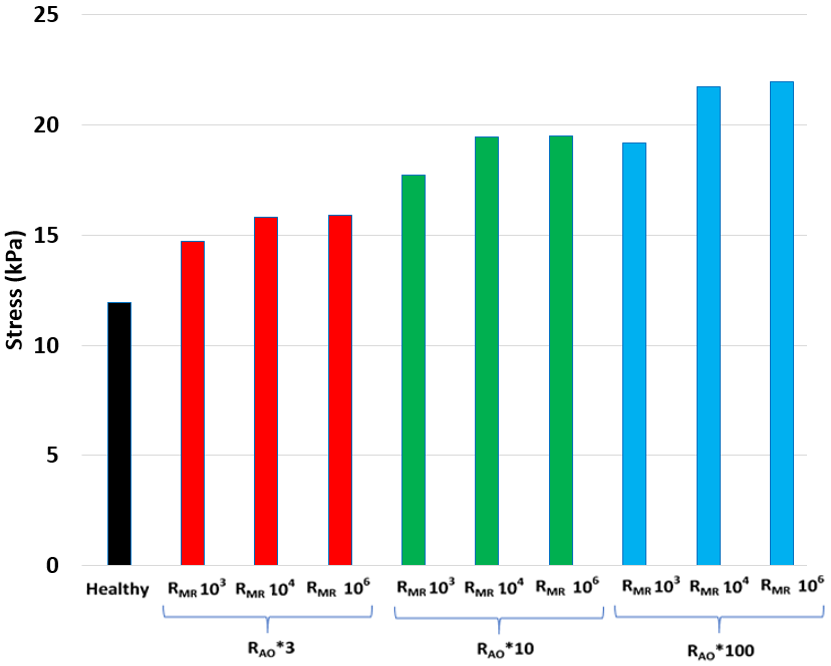


(B)

***Figure S2.*** *Myocardial stress in the fiber direction for the 22 weeks fetal heart, plotted as (A) time-varying waveform, and (B) peak stress, for the healthy and various diseased conditions.*


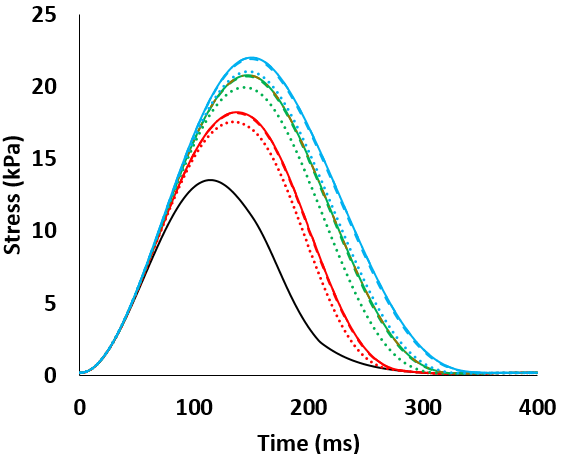


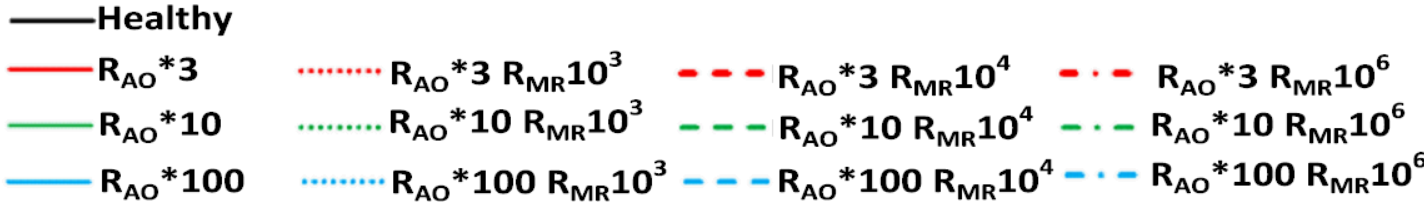


(A)


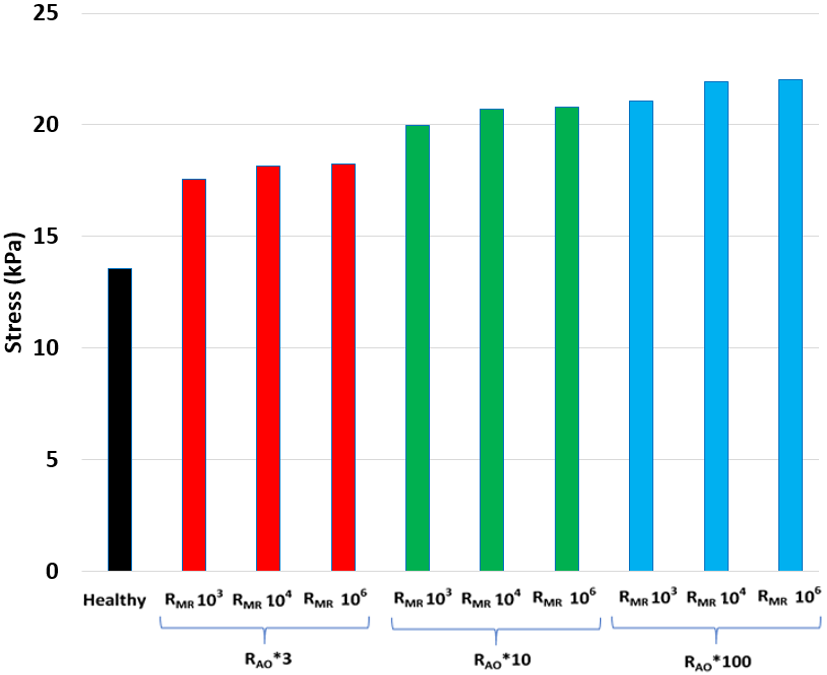


(B)

***Figure S3.*** *Myocardial stress in the fiber direction for the 28 weeks fetal heart, plotted as (A) time-varying waveform, and (B) peak stress, for the healthy and various diseased conditions.*

|  | 22 weeks | 28 weeks | 32 weeks |
| --- | --- | --- | --- |
| Early systole | 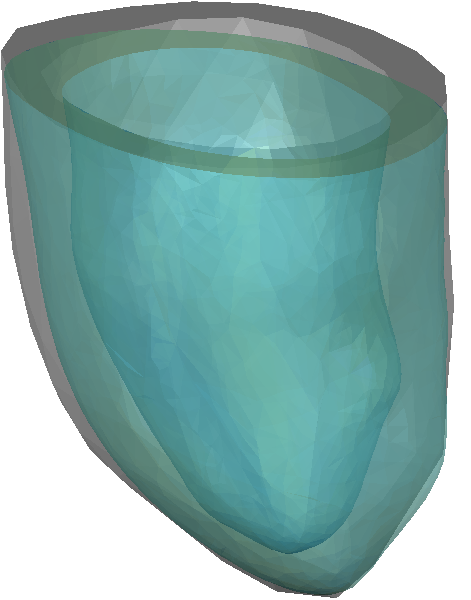  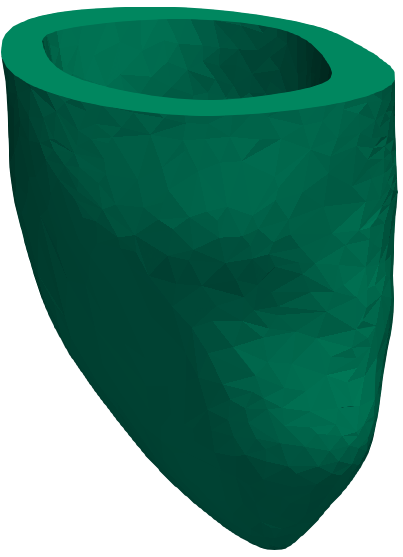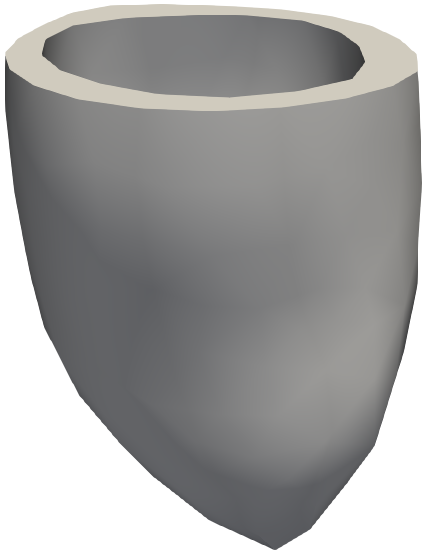 | 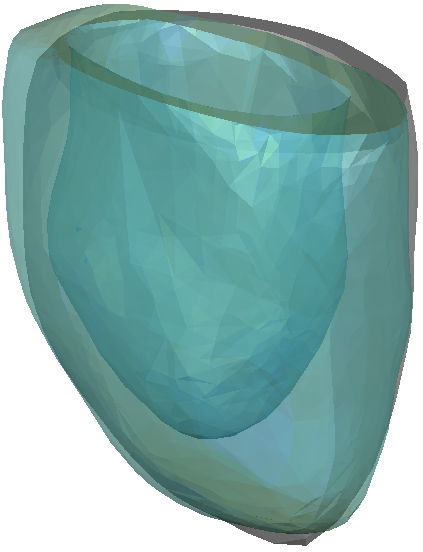  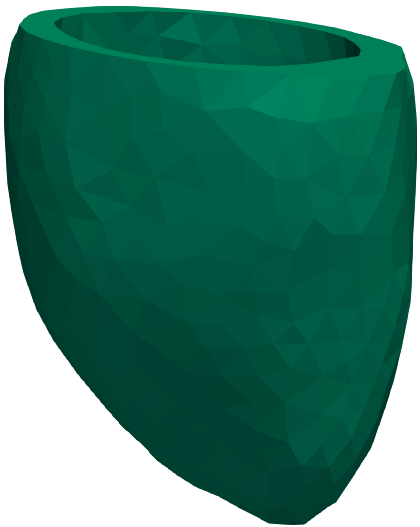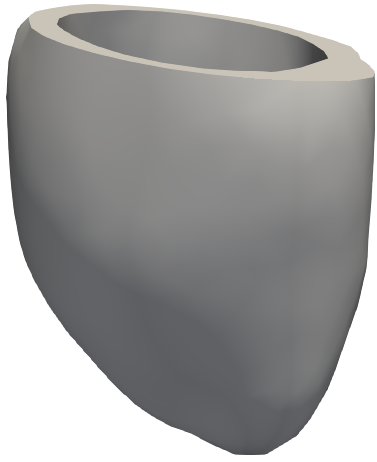 | 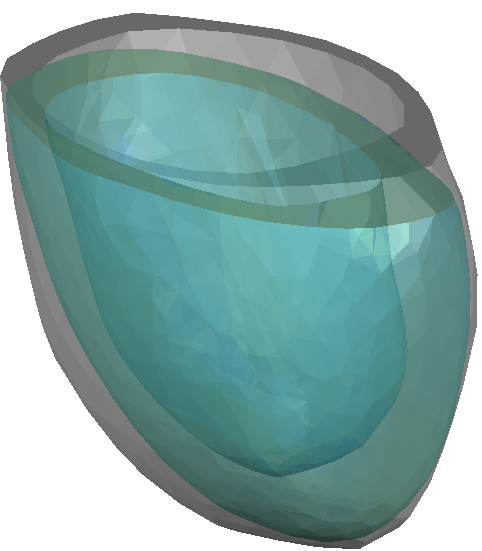  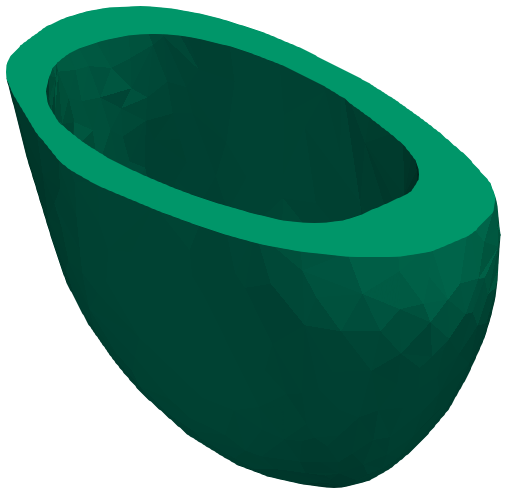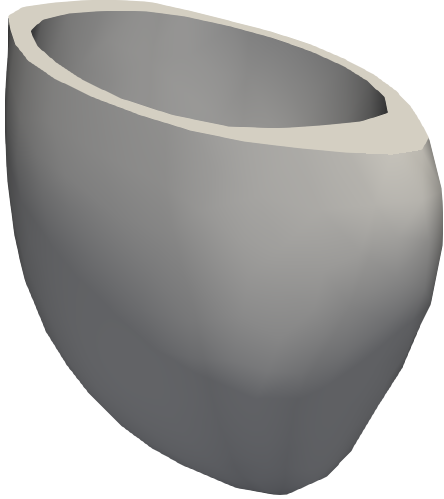 |
| Mid systole | 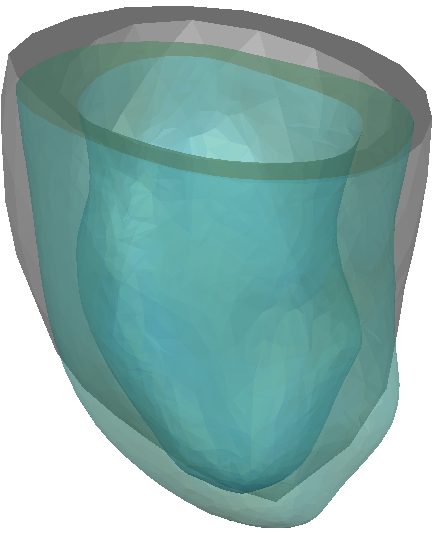  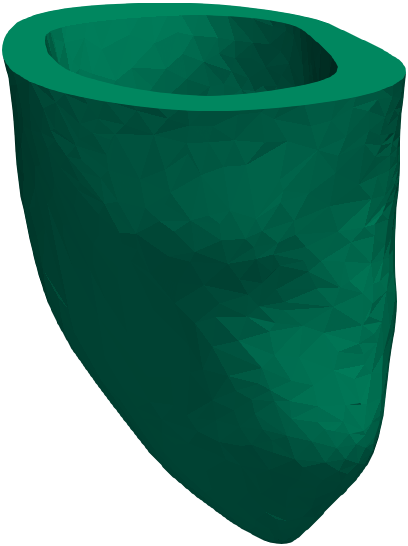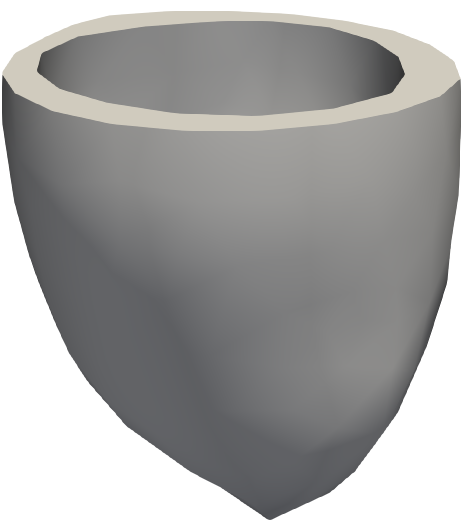 | 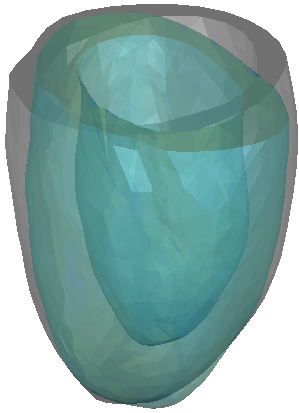  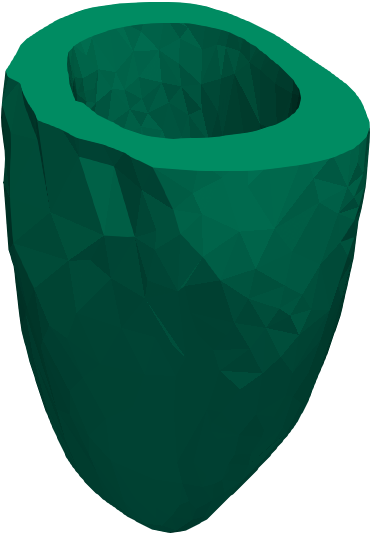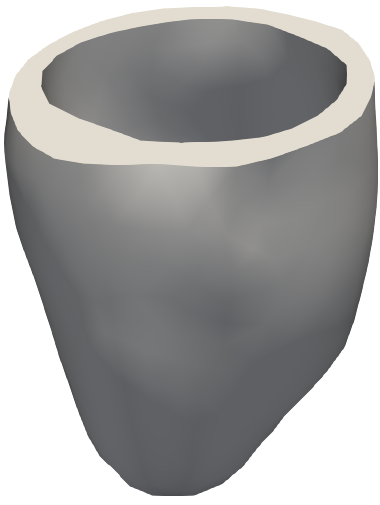 | 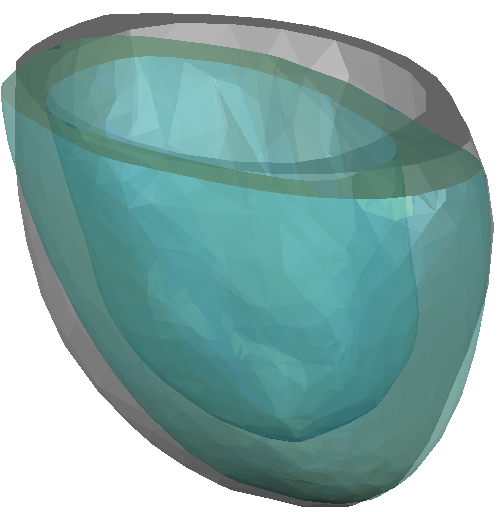 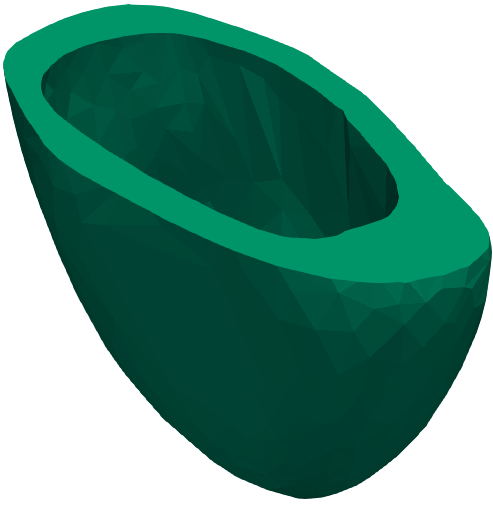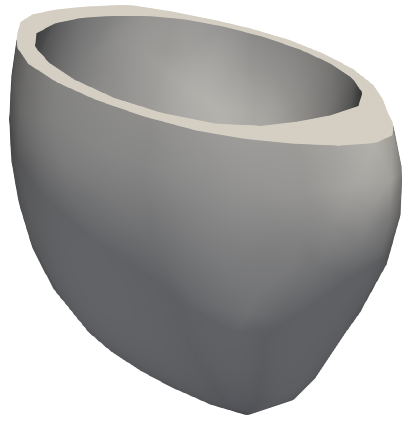 |
| Late systole | 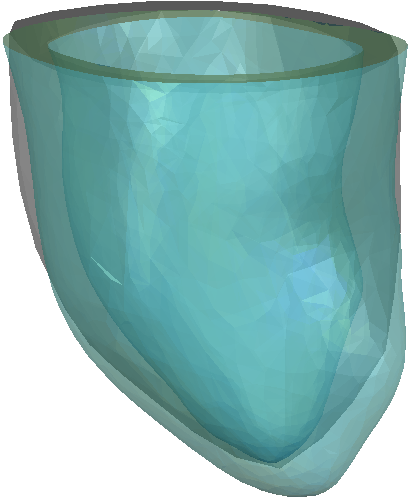  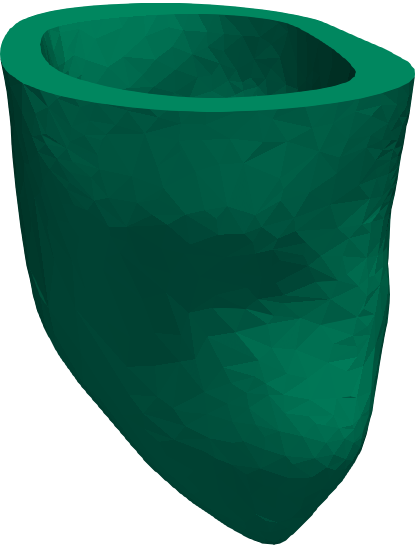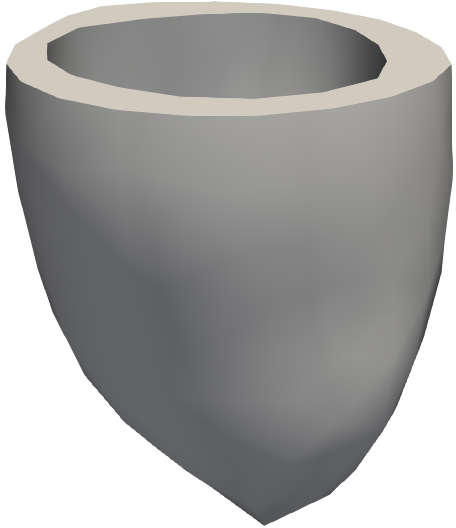 | 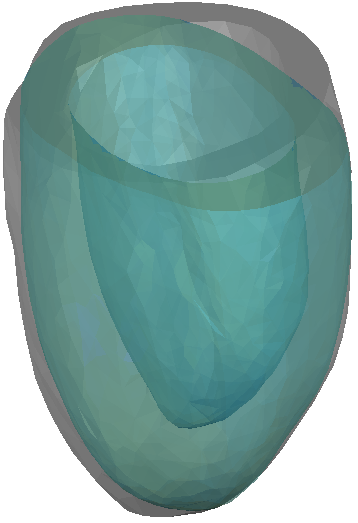  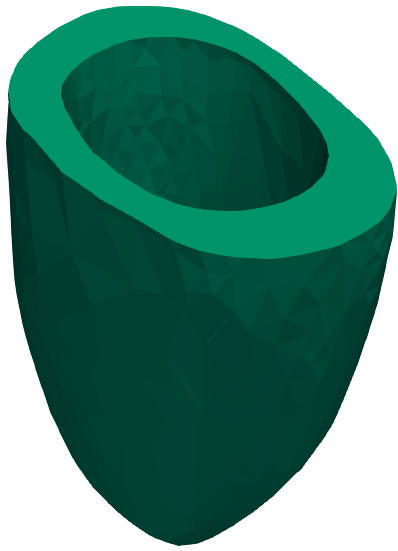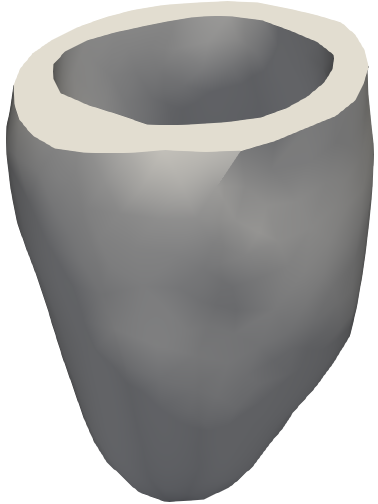 | 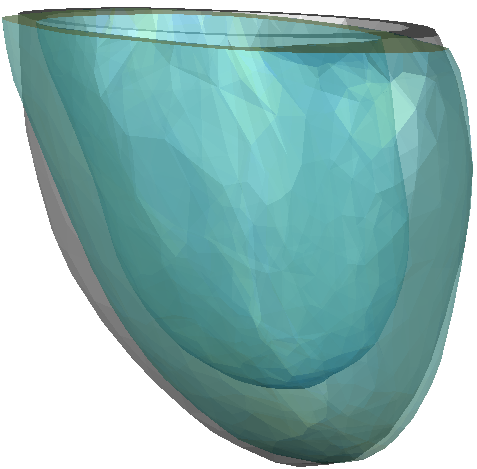  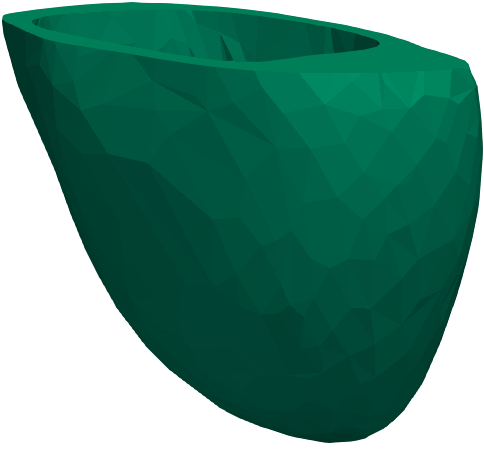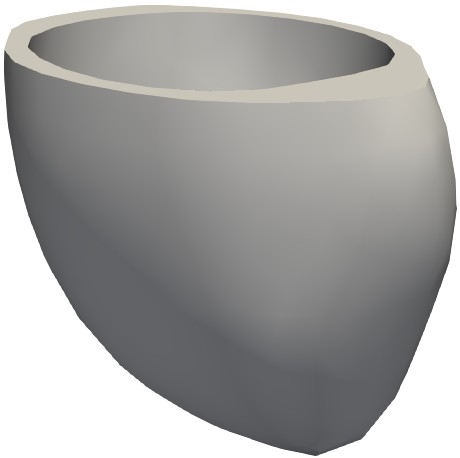 |
| Early Diastole | 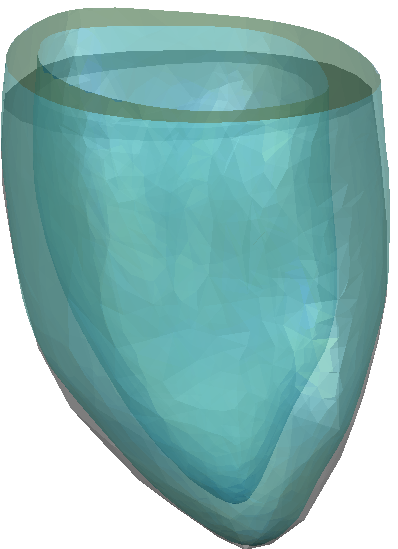  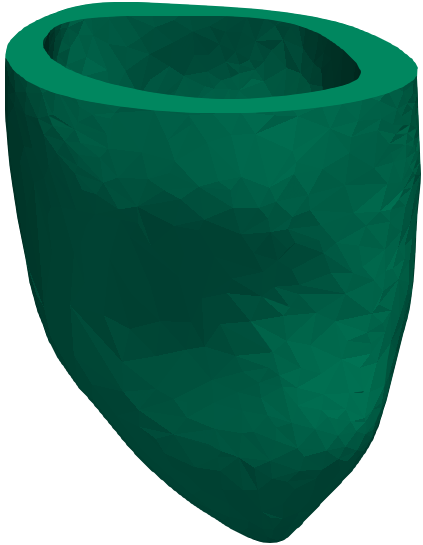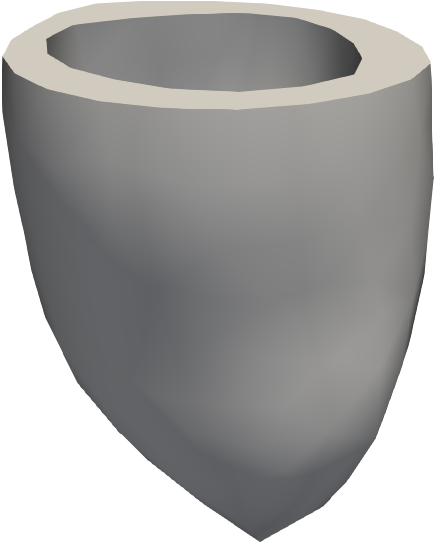 | 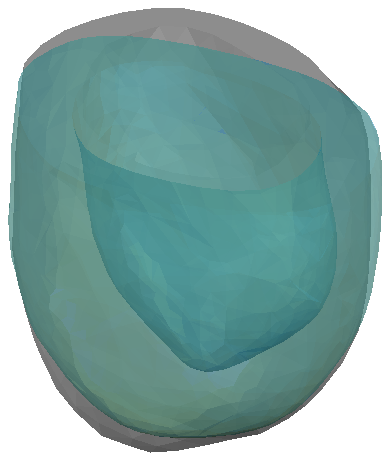  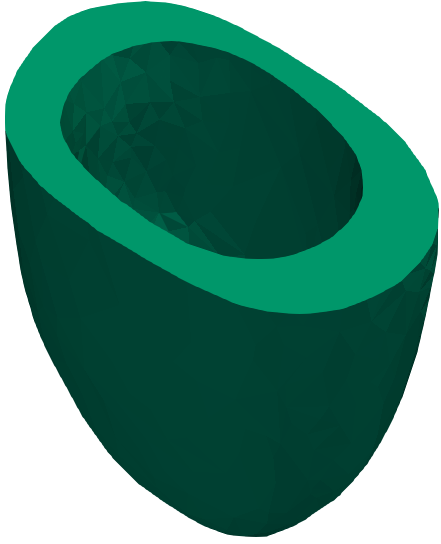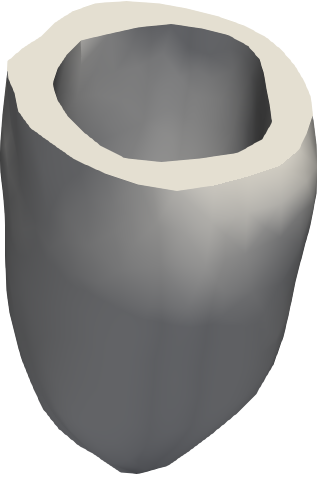 | 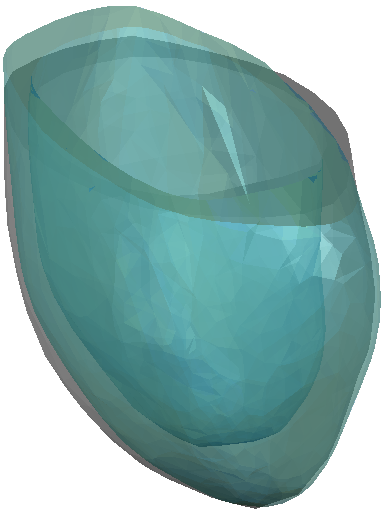  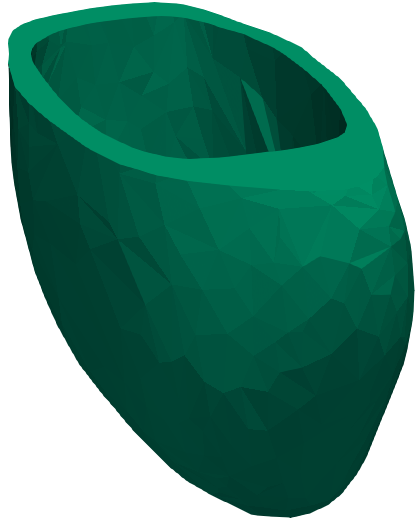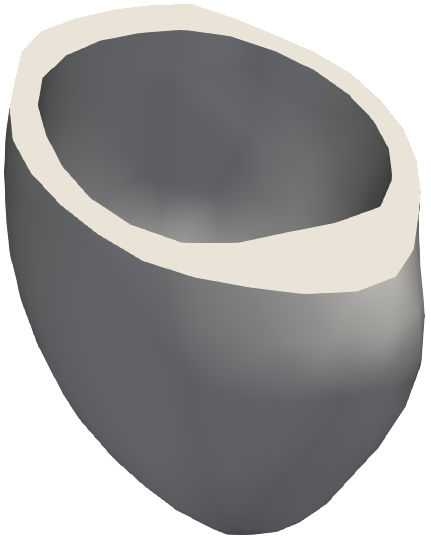 |
| Mid diastole | 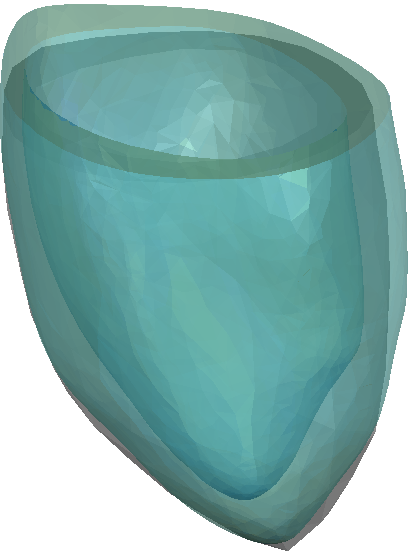  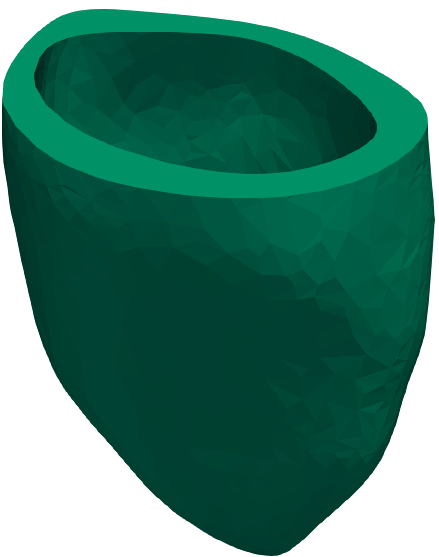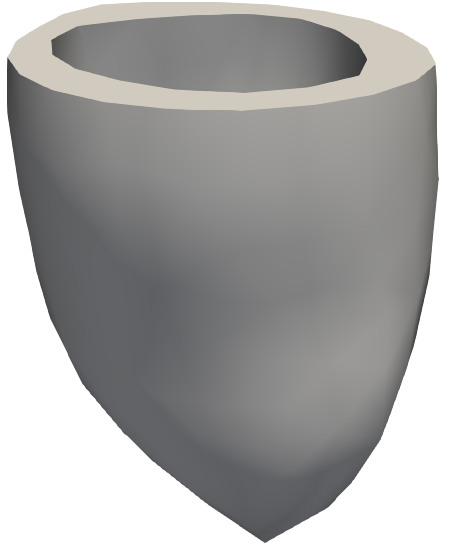 | 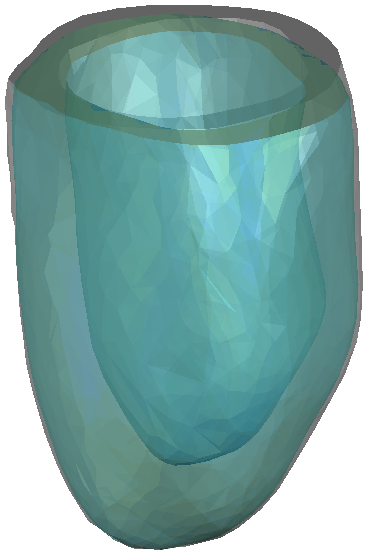  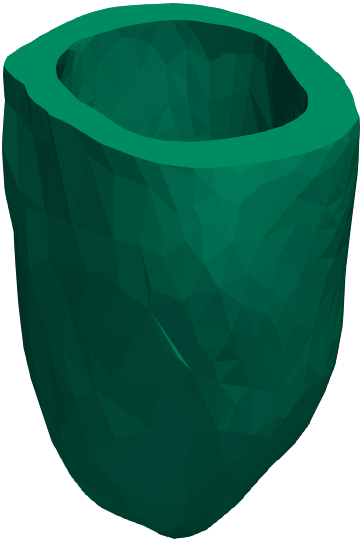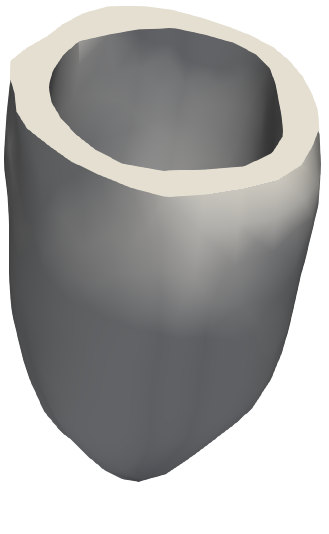 | 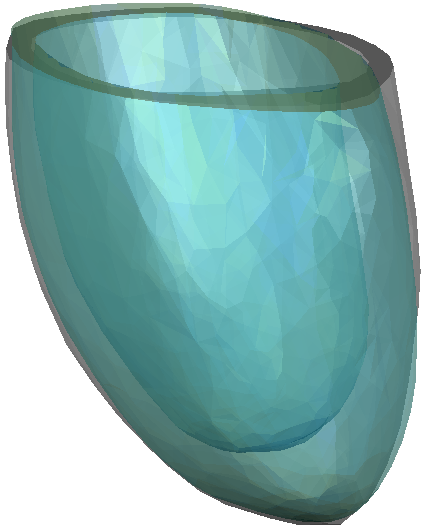  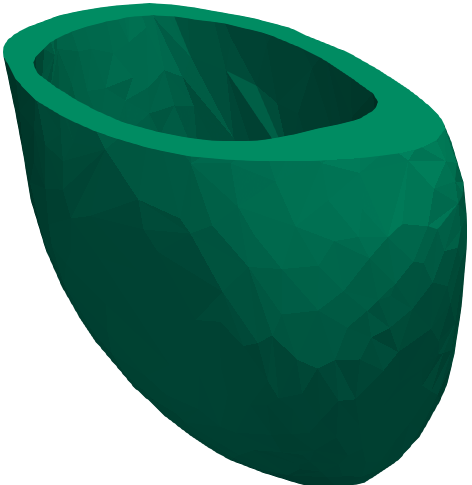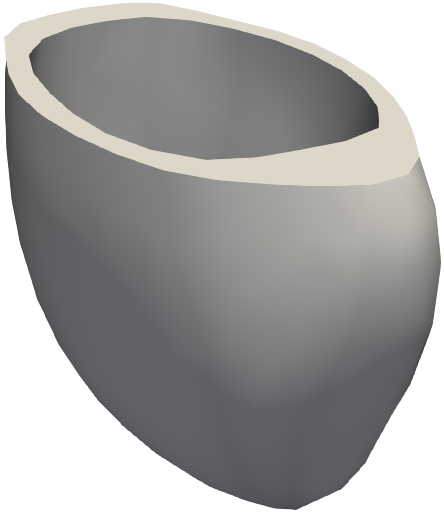 |

***Figure S4.*** *The geometries of the 3 healthy fetal hearts obtained from FE modelling (green), and from ultrasound image motion tracking (gray) at 5 time points across the cardiac cycle, demonstrated in a superimposed manner and individually. The FE and ultrasound geometries showed a good match.*

| **(A)** | 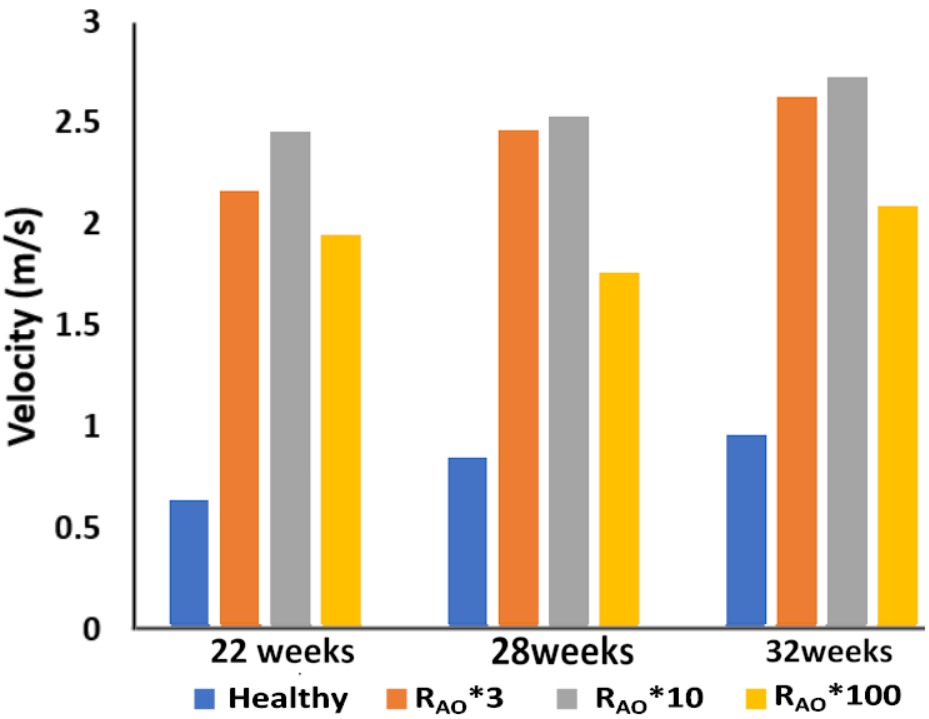 |
| --- | --- |
| **(B)** | 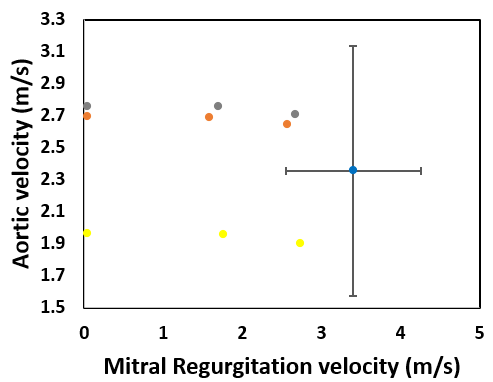 |

***Figure S5.*** *Plot of data in Table 2 of the manuscript. Peak flow velocities (m/s) at the valves of fetal hearts (A) for the aortic stenosis diseased FE simulation scenarios, compared to healthy hearts, and (B) for the aortic stenosis and mitral regurgitation FE simulation scenarios, compared to data from our disease clinical cohort (plotted with standard deviations).*


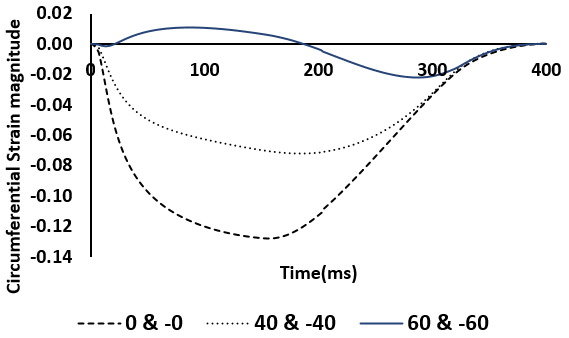

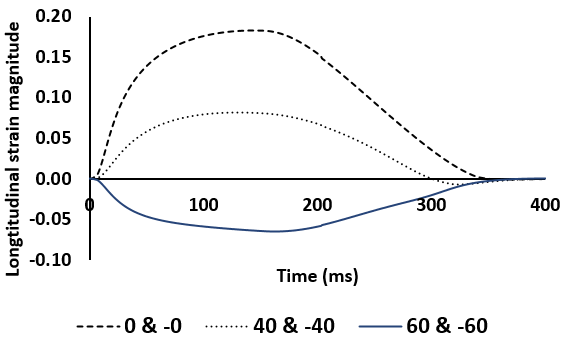


***Figure S6.*** *Experimental simulations of various fiber orientation configurations (epi- & endo- fiber angles) using the baseline case of R_AO_*100, R_MR_10^4^. In the 0^o^ & 0^o^ and 40^o^ & -40^o^ cases, insufficient fibers aligned in the longitudinal direction resulted in unnatural longitudinal stretch.*

***Table S4.*** *Valve flow velocities, calculated based on discharge coefficient formulation, for various aortic stenosis and mitral regurgitation cases for the 22 weeks fetal heart.* AV: aortic valve MV: mitral valve

|  |  |  |  |  |  |  |  |  |  |  |  |  |
| --- | --- | --- | --- | --- | --- | --- | --- | --- | --- | --- | --- | --- |
|  | R_AO_*3 | | | | R_AO_*10 | | | | R_AO_*100 | | | |
| R_MR_10^3^ | AV: | 2.139 | MV: | 2.709 | AV: | 2.437 | MV: | 2.709 | AV: | 2.069 | MV: | 2.709 |
| R_MR_10^4^ | AV: | 2.212 | MV: | 1.737 | AV: | 2.537 | MV: | 1.737 | AV: | 2.217 | MV: | 1.751 |
| R_MR_10^6^ | AV: | 2.221 | MV: | 0.04 | AV: | 2.548 | MV: | 0.04 | AV: | 2.217 | MV: | 0.04 |
|  |  |  |  |  |  |  |  |  |  |  |  |  |
|  |  |  |  |  |  |  |  |  |  |  |  |  |
| ***Table S5.*** *Valve flow velocities, calculated based on discharge coefficient formulation, for various aortic stenosis and mitral regurgitation cases for the 32 weeks fetal heart.* AV: aortic valve MV: mitral valve | | | | | | | | | | | | |
|  |  |  |  |  |  |  |  |  |  |  |  |  |
|  | R_AO_*3 | | | | R_AO_*10 | | | | R_AO_*100 | | | |
| R_MR_10^3^ | AV: | 2.663 | MV: | 2.724 | AV: | 2.8 | MV: | 2.828 | AV: | 2.57 | MV: | 2.878 |
| R_MR_10^4^ | AV: | 2.704 | MV: | 2.342 | AV: | 2.847 | MV: | 2.465 | AV: | 2.639 | MV: | 2.529 |
| R_MR_10^6^ | AV: | 2.712 | MV: | 0.131 | AV: | 2.862 | MV: | 0.147 | AV: | 2.647 | MV: | 0.155 |

**References**

1. Shavik S. M., S. T. Wall, J. Sundnes, D. Burkhoff and L. C. Lee. Organ‐level validation of a cross‐bridge cycling descriptor in a left ventricular finite element model: effects of ventricular loading on myocardial strains. *Physiological Reports* 5: e13392, 2017.

2. Zhou S., L. Xu, L. Hao, H. Xiao, Y. Yao, L. Qi and Y. Yao. A review on low-dimensional physics-based models of systemic arteries: application to estimation of central aortic pressure. *Biomedical engineering online* 18: 41, 2019.
